# Altered metabolic profiles of dermatomyositis with different myositis-specific autoantibodies associated with clinical phenotype

**DOI:** 10.3389/fimmu.2024.1429010

**Published:** 2024-11-25

**Authors:** Nan Wang, Lili Shang, Zhaojun Liang, Min Feng, Yanlin Wang, Chong Gao, Jing Luo

**Affiliations:** ^1^ Department of Rheumatology, The Second Hospital of Shanxi Medical University, Taiyuan, Shanxi, China; ^2^ Shanxi Key Laboratory of Rheumatism Immune Microecology, The Second Hospital of Shanxi Medical University, Taiyuan, Shanxi, China; ^3^ Second Clinical Medical College, The Shanxi Medical University, Taiyuan, Shanxi, China; ^4^ Department of Pathology, Brigham and Women’s Hospital, Harvard Medical School, Boston, MA, United States

**Keywords:** dermatomyositis, metabolomics, biomarkers, interstitial lung disease, anti-MDA5, anti-TIF1-γ, anti-Jo-1

## Abstract

**Introduction:**

Dermatomyositis (DM) is an idiopathic inflammatory myopathy. Because of clinical heterogeneity, the metabolite profile of DM patients with different myositis-specific autoantibodies (MSAs) remains elusive. This study aimed to explore the metabolomics characteristics of the serum in DM with different MSAs, low or high disease activity, and interstitial lung disease.

**Methods:**

Untargeted metabolomics profiling was performed in the serum of a discovery cohort (n=96) and a validation cohort (n=40), consisting of DM patients with MSAs, low or high disease activity, and/or interstitial lung disease (DM-ILD) compared to age- and gender-matched healthy controls (HCs).

**Results:**

The lipid profile in DM was found to be abnormal, especially dysregulated glycerophospholipid metabolism and fatty acid oxidation, which might affect the pathogenesis of DM by disrupting the balance of Th17 and Treg. We identified potential biomarkers of DM that can distinguish between low or high disease activity and reflect lung involvement. Two metabolite combinations including pro-leu, FA 14:0;O can distinguish high disease activity DM from low disease activity DM and HCs, and five including indole-3-lactic acid, dihydrosphingosine, SM 32:1;O2, NAE 17:1, and cholic acid can distinguish DM-ILD from DM without ILD (DM-nonILD). DM with different MSAs had unique metabolic characteristics, which can distinguish between MDA5+DM, Jo-1+DM, and TIF1-γ+DM, and from the antibody-negative groups. The sphingosine metabolism has been found to play an important role in MDA5+DM, which was associated with the occurrence of ILD.

**Discussion:**

Altered metabolic profiles of dermatomyositis were associated with different myositisspecific autoantibodies, disease activity, and interstitial lung disease, which can help in the early diagnosis, prognosis, or selection of new therapeutic targets for DM.

## Introduction

Dermatomyositis (DM) is a rare systemic immune-mediated inflammatory myopathy, which is heterogeneous in the clinic. Besides the skin, it also involves important organs such as the lung, and the severity of DM is related to the type of organ involvement (1). Patients with DM often present with interstitial lung disease (ILD), with a prevalence of approximately 40% (2–4). Importantly, ILD has the most severe extramuscular involvement in DM, which is deeply related to a reduced quality of life and worse prognosis (3, 5). Therefore, early diagnosis is essential to prevent irreversible organ damage with DM progression.

Metabolic changes in the body are downstream of genes and proteins, reflecting the biological phenotype. The discovery of distinct DM autoantibodies and their correlation with specific clinical phenotypes have transformed patient categorization (6), especially myositis-specific antibodies (MSAs). Whether and how each autoantibody influences downstream metabolic processes of disease has rarely been studied. MSAs, including anti-Mi2, anti-MDA5, anti-NXP2, anti-TIF1-γ, and anti-SAE antibodies, may be associated with different DM subtypes in terms of skin manifestations, systemic involvement, and cancer risk (7). For example, muscle disease and arthritis are more common in patients with anti-Jo-1 antibodies (8), and tumors are more common in patients with anti-TIF1-γ (9); however, patients with anti-MDA5 autoantibodies can develop rapidly progressive ILD (2, 3, 5, 10), and their related mortality is very high.

As a new system biology method, metabolomics is increasingly used to evaluate metabolic disorders in human diseases, which has a good prospect of finding new disease biomarkers, clinical diagnosis, and efficacy prediction (11, 12). A study based on an untargeted metabolomic approach found that glutamine, methionine, isoleucine, tryptophan, glutamate, indole, protocatechuic acid, and phenylalanine were potential biomarkers for the diagnosis of DM in terms of sensitivity and specificity (13). Some studies have also found that abnormal lipid changes through metabolomics had a potential role in the diagnosis and treatment of DM (14, 15). These studies implied that metabolomics might be a potentially critical means in the future in terms of early diagnosis and novel therapeutic targets of DM. However, the research of metabolomics and lipidomics on disease activity, organ involvement, and antibody typing for the diagnosis of DM is limited.

In this study, non-targeted metabolomics was used to analyze the serum metabolic profile of DM. Univariate analysis, multivariate statistical analysis, and machine learning models were used to screen key metabolites and identify potential biomarkers of DM with unique MSAs, which can reflect disease activity and lung involvement. Meanwhile, the metabolic characteristics of anti-MDA5, anti-TIF1-γ, and anti-Jo-1 positive DM were studied to explore the key metabolic pathways that promote the development of the disease. These results are helpful to understand the occurrence and development of DM at the molecular level and to realize the early diagnosis, prognosis, and targeted therapy of DM.

## Materials and methods

### Patients and serum sample collection

Between January 2016 and July 2021, 96 participants [67 patients with DM and 29 healthy controls (HCs)] were assigned to the discovery cohort to evaluate biomarkers, and 40 participants (28 with DM and 12 HCs) were assigned to the validation cohort to test candidate biomarkers. All patients were in accord with the American College of Rheumatology (ACR) classification criteria for DM without the history of other autoimmune diseases. HCs with matched age and gender were enrolled at the Second Hospital of Shanxi Medical University. HCs also had no history of autoimmune diseases. Subjects in any one or more of the following categories were excluded from our analysis: (1) the presence of type I or II diabetes, (2) active viral and/or bacterial infection, and (3) received high-dose glucocorticoid pulse therapy. The disease activity of DM was evaluated using the Myositis Disease Activity Assessment Visual Analogue Scales (MYOACT), which was established by the International Myositis Assessment and Clinical Studies (IMACS) group, including constitutional, cutaneous, skeletal, gastrointestinal, pulmonary, cardiovascular, muscle, extramuscular, and a global score. ILD was diagnosed by a rheumatologist and radiologist based on HRCT-revealed reticular abnormalities and honeycombing and clinical features. This study was approved by the ethics committee of the Second Hospital of Shanxi Medical University (2019YX266).

Clinical data on comorbidities and therapy that may affect metabolism profiles were retrieved by retrospective review of patients’ records. None of the 95 DM patients had diabetes mellitus. In the discovery cohort, four patients of DM were diagnosed with hypothyroidism and one patient was combined with hyperthyroidism, while only one DM patient was combined with hypothyroidism in the validation cohort. The rest of the patients had normal serum levels of thyroid-stimulating hormone (TSH), and thyroid disease was excluded. In the discovery cohort, 33 patients were on treatment with prednisone with a median daily dose of 15 mg/day. A total of 22 patients had an additional immunosuppressive drug (Methotrexate, thalidomide, leflunomide, or hydroxychloroquine); one patient was treated with hydroxychloroquine only, and 33 patients were newly diagnosed with DM or untreated. In the validation cohort, 12 patients were on treatment with prednisone, and 16 patients were newly diagnosed with DM or untreated at the time of serum sampling. None of DM patients took carnitine supplements for treatment.

Fasting serum was collected from each subject with the patient’s consent and stored at −80°C until use. Clinical data collection for DM patients was described in the Supplementary methods. The clinical characteristics of the participants are listed in **Table 1**; **Supplementary Table S1**.

**Table 1:** Demographics and clinical characteristics of DM patients and health controls.

Characteristic	Discovery set (n=96)		Validation set (n=40)	
	DM (n=67)	HC (n=29)	DM (n=28)	HC (n=12)
Sex, female (%)	55 (82.09%)	23(79.31%)	22 (78.57%)	10(83.33%)
Age (years)	49.09 ± 15.34	52.48 ± 13.30	49.04 ± 15.92	53.33 ± 13.45
BMI (kg/m^2^, mean ± SD)	24.09 ± 5.27		23.27 ± 3.4	
Age at onset (years)	46.33 ± 15.35		46.04 ± 17.71	
Disease duration (median mouth, IQR)	7 (2, 27)		2.5 (1, 39)	
LDH, U/L	482.63 ± 372.11		462.09 ± 299.03	
Cr, μmol/L	48.55 ± 10.66		49.14 ± 13.84	
CK, μmol/L	1177.12 ± 3670.0		932.14 ± 2123.2	
AST, U/L	83.25 ± 104.73		86.17 ± 98.98	
ALT, U/L	69.37 ± 72		100.81 ± 184.06	
TC, mM/L	4.49 ± 1.23		4.48 ± 0.89	
TG, mM/L	1.94 ± 1.08		1.74 ± 0.82	
HDL-C, mmol/L	1.14 ± 0.35		1.14 ± 0.40	
LDL-C, mmol/L	2.49 ± 0.84		2.6 ± 0.61	
Glucose, mmol/L	5.68 ± 1.55		5.91 ± 2.51	
ESR, mm/h	44.55 ± 28.37		42.29 ± 32.24	
CRP, mg/	29.40 ± 39.26		31.72 ± 61.10	
ANA (1:80), (n, %)	33 (49.25%)		15 (53.57%)	
MYOACT	10.80 ± 4.76		10.86 ± 5.13	
MYOACT_muscle_	3.49 ± 2.23		3.80 ± 2.71	
MYOACT_extramuscular_	7.31 ± 4.08		7.05 ± 3.36	
Skin lesions, n (%)				
Gottron’s papules or sign	25 (37.31%)		14 (50%)	
Heliotrope rash	59 (88.06%)		24 (85.71%)	
Mechanics hand	7 (10.45%)		2 (7.14%)	
Cutaneous ulcerations	9 (13.43%)		3 (10.71%)	
Muscle, n (%)				
Myalgia	26 (38.81%)		8 (28.57%)	
Muscle weakness	53 (79.10%)		22 (78.57%)	
Other, no. (%)				
Hoarseness or sore throat, dysphagia	36 (53.73%)		10 (35.71%)	
Arthralgia	34 (50.75%)		15 (53.57%)	
Fever	33 (49.25%)		10 (35.71%)	
Interstitial lung disease	27 (40.30%)		6 (21.43%)	
Cardiac involvement	10 (14.93%)		0	
Myositis-specific antibodies, no. (%)				
Negative	20 (29.85%)		9 (32.14%)	
MDA-5	9 (13.43%)		2 (7.14%)	
TIF-1γ	8(11.94%)		0	
Jo-1	4 (5.97%)		2 (7.14%)	
Mi-2	2 (2.98%)		1 (3.57%)	
NXP-2	3 (4.48%)		2 (7.14%)	
Ro52	15 (22.39%)		3 (10.71%)	
Treatments				
Prednisone ≤15 mg/day	17 (25.37%)		7 (25%)	
Prednisone >15 mg/day	16 (23.88%)		5 (17.86%)	
csDMARDs	23 (34.33%)		6 (21.43%)	

BMI, body mass index; LDH, lactate dehydrogenase; Cr, creatinine; CK, creatine kinase; AST, aspartate aminotransferase; ALT, alanine aminotransferase; TC, total cholesterol; TG, triglyceride; HDL-C, high density lipoprotein cholesterol; LDL-C, low density lipoprotein cholesterol; ESR, erythrocyte sedimentation rate; CRP, C-reactive protein; MYOACT, myositis disease activity assessment visual analogue scales.

### Untargeted metabolomic profiling

Untargeted metabolic profiles of serum samples from DM patients and HCs were measured by ultra-high-performance liquid chromatography-composed time-of-flight mass spectrometry (UPLC-TOF-MS), and exhaustive metabolite extraction and LC-MS analysis methods were described in Supplementary methods. The raw data were imported to XCMS (version 3.6.3) for automatic data prepossessing including peak picking and retention time correction. Subsequently, the substances with detection rate <50% or relative standard deviation >30% were filtered. Then, the resulting data matrixes were imported into SIMCA 14.0 software (Umetrics, Sweden) for multivariate data analysis, including principal component analysis (PCA) and orthogonal partial least square discriminant analysis (OPLS-DA). The variable importance in the projection (VIP) values from OPLS-DA models, fold change (FC), and *p*-value or false discovery rate (FDR) correction were performed to screen the differential metabolites. The metabolites were identified by OSI/SMMS software (Dalian ChemData Solution Information Technology Co., Ltd., China), MSDIAL (version 5.1.221218), and other online databases, including Human Metabolome Database (http://www.hmdb.ca/), Lipidmaps (https://lipidmaps.org/), and LipidBlast (https://fiehnlab.ucdavis.edu/projects/lipidblast).

### Statistical analysis

Statistical analysis of clinical data and differential metabolites was performed using the SPSS 22.0, GraphPad Prism 8.0, and MetaboAnalyst 5.0. Categorical and quantitative variables were described as frequencies, percentage, mean ± standard deviation, or median (Q25, Q75). Data of demographic and clinical features were compared between groups by the non-parametric Mann–Whitney U-test or independent sample t-test, as appropriate. Correlation analysis was performed using the Spearman or Pearson correlation test. Receiver operating characteristic (ROC) curve analysis was used to evaluate the diagnostic performance of potential biomarkers. The lasso regression was performed by R (glmnet package) for screening metabolites, and partial least squares discrimination analysis (PLS-DA), support vector machines (SVM) classification model, and random forest (RF) model were performed in MetaboAnalyst 5.0 to validate the selected biomarkers.

## Results

### Serum metabolic profiling of DM

**Figure 1:**
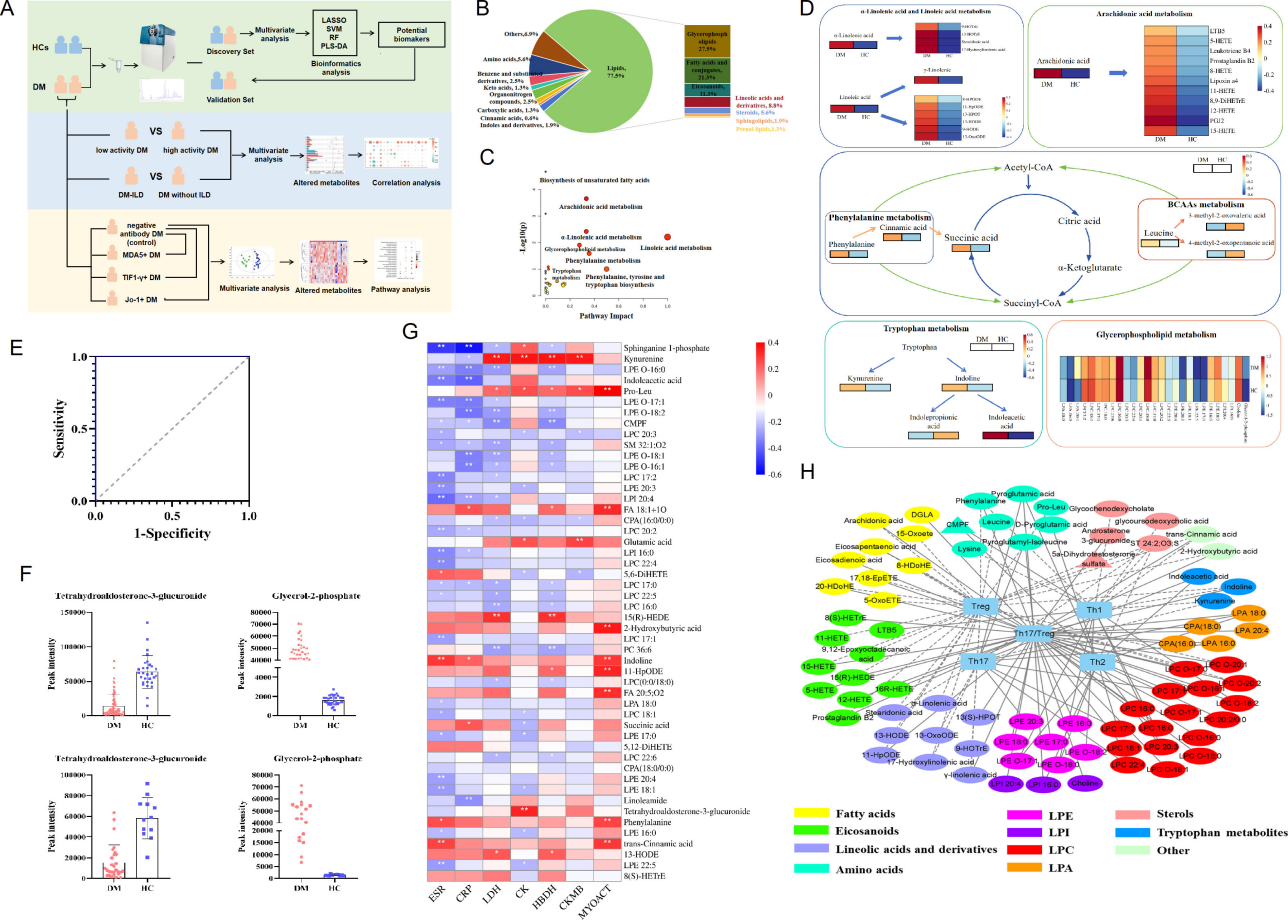
Altered metabolic profiles in the serum of dermatomyositis. **(A)** The workflow of study design and data analysis. **(B)** The composition and proportions of differential metabolites between DM and HCs. **(C)** Pathway analysis of the differentially altered metabolites identified in patients with DM compared with HCs. **(D)** Altered differential metabolite involved in different metabolic pathways. **(E)** Fitting ROC analysis of two biomarkers screened by lasso regression and multiple logistic regression models. **(F)** Distribution of two biomarkers in DM and HC in the discovery cohort. **(G)** Correlation heatmap of differential metabolites and markers of inflammation and disease activity in 95 patients with DM. **(H)** Integrative network of associations reflecting the interactions of differential metabolites and T-cell subpopulation in 95 patients with DM. Network revealed associations (*p*<0.05) between differentially abundant metabolites and T-cell subpopulation in DM. * p< 0.05, ** p< 0.01.

The workflow of research design and data analysis is shown in **Figure 1A**. All the serum samples were analyzed by UPLC-TOF-MS. The PCA model and OPLS-DA model were constructed and showed a significant difference between the DM and HCs (**Supplementary Figures S1**, **S2**). The repeatability of metabolic profiling was evaluated using quality control (QC) samples, indicating that the analytical methods were reliable and acceptable (**Supplementary Figure S1**).

Subsequently, based on the criteria of VIP>1, FDR<0.05, and FC>1.5 or FC<0.67, a total of 160 differential endogenic metabolites were screened between the DM and HC groups. Among them, 17 serum metabolites decreased and 143 increased in the DM group (**Supplementary Table S2**). The validation cohort containing 28 DM and 12 HCs was used to evaluate the reliability of differentially altered metabolites; the PCA model showed the significant separation of the metabolic spectra between patients with DM and HCs in the verification cohort (**Supplementary Figure S3**). These differential metabolic features between DM and HCs mainly include lipids, amino acids, benzene and substituted derivatives, organonitrogen compounds, carboxylic acids, cinnamic acids, indoles and derivatives, and other compounds (**Figure 1B**). Amino acids were detected as the predominant type of polar compounds, while glycerophospholipids and fatty acids were the principal types of lipid compounds.

To further explore the pathways possibly related to DM, 160 differential metabolites were used to perform metabolic pathway analysis between HC and DM groups. As shown in **Figures 1C, D**, for DM, the altered metabolites of the fatty acid metabolic pathway accounted for an important proportion, including α-linolenic acid and linoleic acid metabolism and arachidonic acid metabolism. Linolenic acid, linoleic acid, arachidonic acid, and derived oxidized lipids increased in DM. In addition, there were other metabolic pathways, such as amino acid metabolism, tricarboxylic acid cycle, tryptophan metabolism, and glycerophospholipid metabolism, that have shown important effects on the development of DM.

Then, we used lasso regression to further screen the altered endogenous differential metabolites, with passing 10-fold cross-validation and adjusting the parameters λ. Nine metabolites were identified as potential biomarkers for DM (**Supplementary Table S3**), including tetrahydroaldosterone-3-glucuronide, carnitine, choline, LPC 16:0, α-curcumene, glutamic acid, glycerol-2-phosphate, 2-hydroxybenzothiazole, and DG 41:10. To further improve the diagnostic efficiency of DM, the forward stepwise regression model was performed, and a panel consisting of tetrahydroaldosterone-3-glucuronide and glycerol-2-phosphate showed that high specificity and sensitivity with the area under the curve (AUC) was 1.0 (**Figure 1E**). The discriminant ability of two biomarkers in the discovery cohort and the validation cohort were analyzed using RF, SVM, and PLS-DA models. The results showed that the accuracy of the two biomarkers in distinguishing the HC and DM groups reached more than 90% in the three models (**Supplementary Figure S4**), and the changing trend of the two biomarkers in the validation cohort was consistent with that in the discovery cohort (**Figure 1F**). To sum up, the serum endogenous metabolites, tetrahydroaldosterone-3-glucuronide and glycerol-2-phosphate, had good discrimination ability for DM.

### Correlation between the DM differential metabolites and clinical features

To explore the possible impact of altered metabolites on disease development, we analyzed the correlation between these metabolites and the clinical features of DM. The results showed that the elevated metabolites in the serum of DM patients were related to disease activity (**Figure 1G**); for example, pro-leu, 2-hydroxybutyric acid, phenylalanine, indoline, trans-cinnamic acid, and 11-HpODE were positively correlated with MYOACT (R=0.2–0.39, *p*<0.05). Kynurenine, pro-leu, glutamic acid, and tetrahydroaldosterone-3-glucuronide were weakly associated with muscle enzyme indexes CK, CKMB, LDH, and HBDH (R=0.2–0.39, *p*<0.05), while sphinganine 1-phosphate, indoleacetic acid, and LPE were negatively correlated with inflammatory indexes (ESR and CRP).

In addition, previous research had found that the number of CD4 + T-cell subsets in the DM was imbalanced, especially the decrease in peripheral Treg cells and increase in Th17/Treg ratio (16). In our study, multiple metabolites were found to be weakly associated with CD4+T cells subsets (|R|>0.2, *p*<0.05, **Figure 1H**). Increased oxidized lipids and amino acids in DM were negatively correlated with the absolute number of Treg, while increased LPC and amino acids were positively correlated with the absolute number of Th17. Linoleic acid and derivatives, arachidonic acid and derivatives, amino acids, glycerophospholipids, and indoles were related to the ratio of Th17/Treg.

### Serum metabolic profiling associated with disease activity of DM

**Figure 2:**
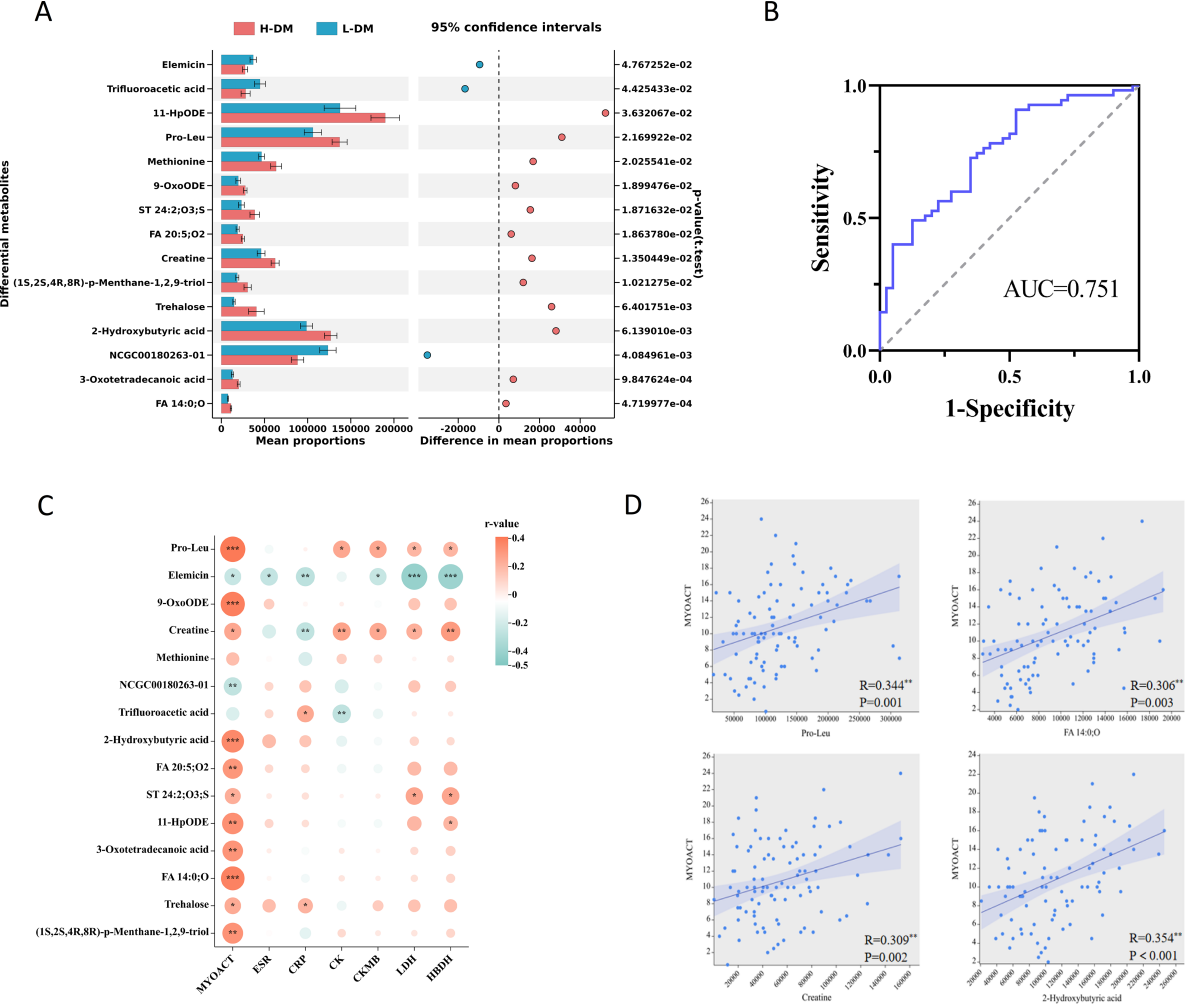
Analysis of serum differential metabolites between low activity DM (L-DM) and high activity DM (H-DM). **(A)** Comparison of the abundance of 15 differentially altered metabolites between L-DM and H-DM. **(B)** ROC curves for differentiating L-DM and H-DM based on combined pro-leu and FA 14:0;O. **(C)** Correlation heatmap of differential metabolites (L-DM vs. H-DM) and clinical features. **(D)** Linear correlation between key metabolites (pro-Leu, FA 14:0;O, creatine and 2-hydroxybutyric acid) and MYOACT. * p< 0.05, ** p< 0.01, *** p< 0.001.

DM was divided into low disease activity DM (L-DM, MYOACT<10) and high disease activity DM groups (H-DM, MYOACT≥10) according to the median MYOACT of all patients with DM. The demographics and clinical characteristics of the two groups are listed in **Supplementary Table S4**. The OPLS-DA models of metabolome showed differences between L-DM and H-DM in positive and negative ions models (**Supplementary Figure S5**). Subsequently, a total of 15 differential endogenous metabolites based on the criteria of VIP>1, *p*<0.05, and FC>1.2 or FC<0.8 were determined between L-DM and H-DM; 13 differential metabolites were enriched in H-DM and two were enriched in L-DM (**Figure 2A**). To distinguish H-DM from L-DM and HCs, the methods of the forward stepwise regression were used to select the altered metabolites. A panel consisting of pro-Leu and FA 14:0;O showed the best predictive efficiency with AUC of 0.751 in the combined for ROC analysis (**Figure 2B**).

Furthermore, we analyzed the correlation between differential metabolites of L-DM and H-DM groups and disease activity in all DM patients using the Spearman’s correlation (**Figure 2C**). The results showed that pro-leu, 9-OxoODE, 2-hydroxybutyric acid, FA 14:0;O, FA 20:5;O2, and 11-HpODE were significantly and positively correlated with MYOACT of DM (R=0.2–0.39, *p*<0.01). Meanwhile, pro-leu and creatine were weakly and positively related to muscle enzyme indices (CK, CKMB, LDH, and HBDH), while elemicin was moderately and negatively related to the LHD and HBDH (|R|=0.4–0.5, *p*<0.001). Linear regression results (**Figure 2D**) indicated that pro-leu, FA 14:0;O, creatine, and 2-hydroxybutyric acid had certain positive linear correlation with MYOACT of DM, implying that the four metabolites might play roles in promoting the occurrence and development of DM. Additionally, creatine levels were positively associated with muscle disease activity (MYOACT_muscle_, R=0.369, *p*<0.001) in DM patients. Serum creatinine (Cr) is the primary metabolite of creatine in muscle, who were negatively correlated with MYOACT_muscle_ in DM patients (R=−0.274, *p*=0.008), although not with MYOACT.

In addition, the absolute number of peripheral Treg cells in the H-DM group was significantly lower than that in the L-DM group (**Supplementary Table S4**). Meanwhile, we found that elevated metabolites in H-DM, such as 2-hydroxybutyric acid, 11-HpODE, 9-OxoODE, and FA 20:5;O2, were significantly negatively correlated with the absolute number of Treg (**Supplementary Figure S6**). These results suggested that the altered metabolites might accelerate the progression of DM by inhibiting the number of Tregs.

### Serum metabolic profiling of DM-associated interstitial lung disease

Interstitial lung disease (ILD) is a common complication of DM and is associated with increased mortality. To investigate biomarkers of DM-associated with ILD (DM-ILD), we divided all DM patients into the DM-ILD group and DM without ILD (DM-nonILD) group according to clinical phenotypes. The demographics and clinical characteristics of the two groups are listed in **Supplementary Table S5**.

**Figure 3:**
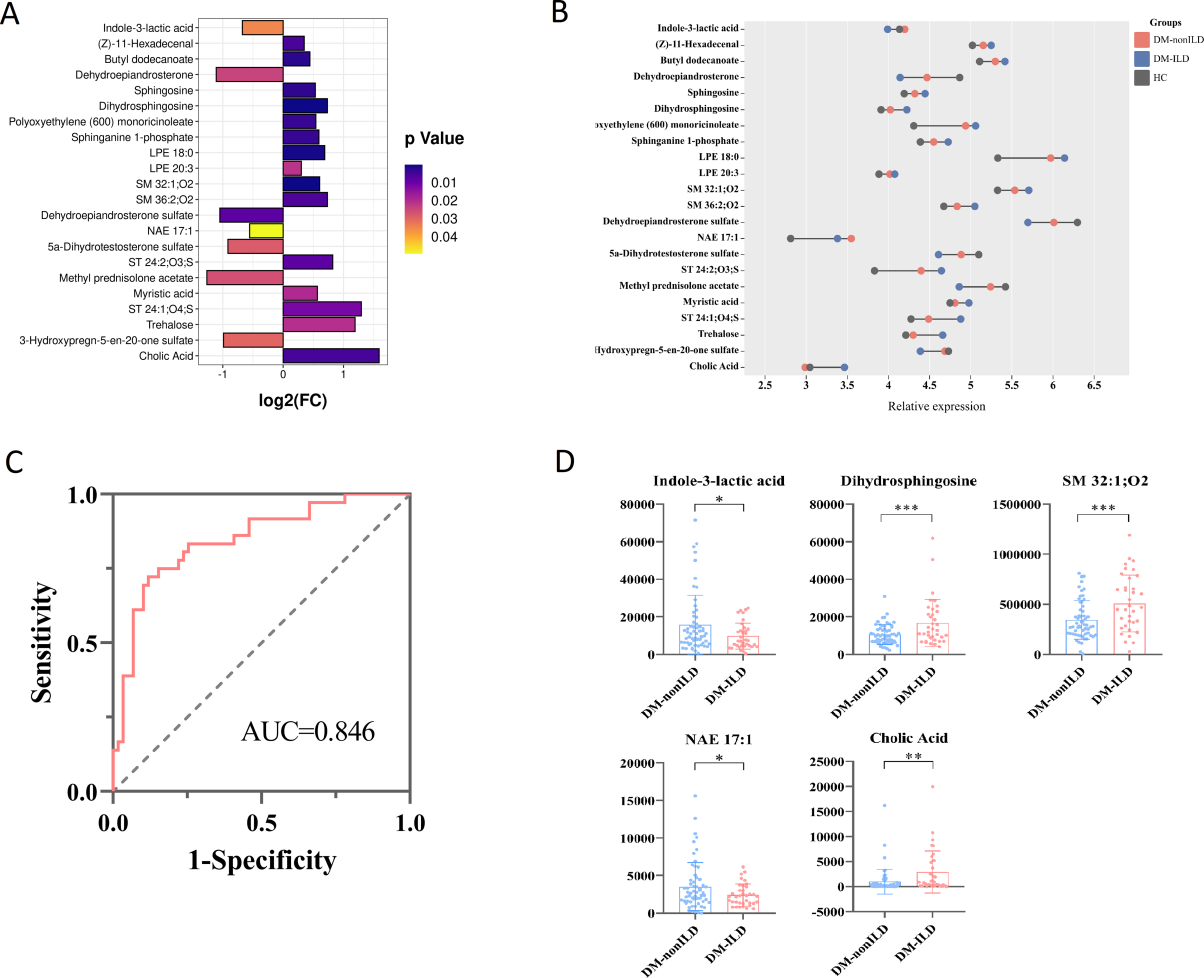
Analysis of serum differential metabolites between DM-ILD and DM-nonILD. **(A)** Bar graph reflecting the fold change (FC) of differential metabolites between DM-ILD and DM-nonILD. **(B)** Dumbbell plot of the distribution of altered metabolite expression levels in the DM-ILD, DM-nonILD, and HCs groups. **(C)** ROC curves for differentiating DM-ILD and DM-nonILD based on combined indole-3-lactic acid, dihydrosphingosine, SM 32:1;O2, NAE 17:1, and cholic acid. **(D)** Scatter plot presenting levels of indole-3-lactic acid, dihydrosphingosine, SM 32:1;O2, NAE 17:1, and cholic acid between DM-ILD and DM-nonILD. * p< 0.05, ** p< 0.01, *** p< 0.001.

In the OPLS-DA models of the metabolome, DM-ILD and DM-nonILD groups could be separated at positive and negative ions models, and 200 permutations were tested and the discriminant models did not overfit (**Supplementary Figure S7**). Subsequently, a total of 22 differential metabolites based on the criteria of VIP>1, *p*<0.05, and FC>1.2 or FC<0.8 were determined between DM-ILD and DM-nonILD, of which seven were decreased and 15 were increased in DM-ILD group (**Figure 3A**).

The variation in differential metabolites in DM-ILD was greater than in DM-nonILD as compared to HCs (**Figure 3B**). To distinguish DM-ILD from DM-nonILD, the method of forward stepwise regression was used to select the potential biomarkers. A panel consisting of indole-3-lactic acid, dihydrosphingosine, SM 32:1;O2, NAE 17:1, and cholic acid showed the best predictive efficiency with AUC of 0.846 in the combined ROC analysis (**Figure 3C**). The distributions of five biomarkers are shown in **Figure 3D**, indole-3-lactic acid and NAE 17:1 was reduced in DM-ILD, while dihydrosphingosine, SM 32:1; O2, and cholic acid increased in DM-ILD.

### Characteristic metabolites in different antibody-positive DM

When comparing the clinical characteristics of low- and high-activity DM or DM-ILD and DM-nonILD, it was found that the positive rate of myositis-specific autoantibodies (MSAs) in H-DM or DM-ILD was higher than that in L-DM or DM-nonILD (**Supplementary Tables S4**, **S5**). We studied the metabolic characteristics of DM with different antibody to explore MSAs influence and role on the disease progression. We tested the serum antibodies of all participants and screened out the antibody-negative DM group (control, n=21), the anti-MDA5+DM group (MDA5+DM, n=10), the anti-TIF1-γ+DM group (TIF1-γ+DM, n=8), and the anti-Jo-1+DM group (Jo-1+DM, n=6). The demographics and clinical characteristics of four groups are shown in **Supplementary Table S6**. The metabolic profile of each antibody-positive group was respectively compared with the control group by the OPLS-DA model, suggesting that MDA5+DM, TIF1-γ+DM, Jo-1+DM, and control groups could be separated, respectively (**Supplementary Figures S8**–**S10**).

**Figure 4:**
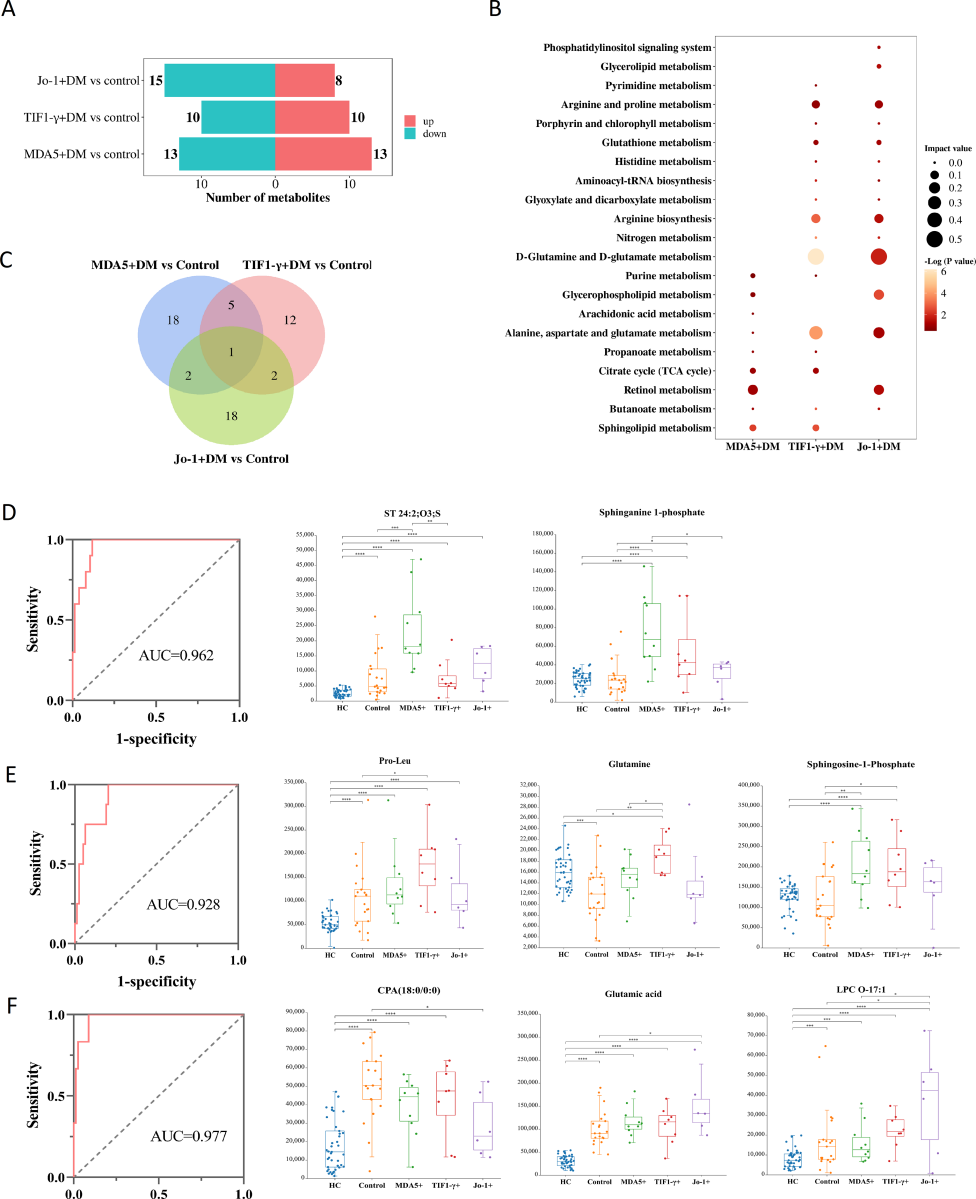
Specific metabolites in serum of DM with different antibody positive [MDA5+DM, TIF1-γ+DM, Jo-1+DM and antibody negative DM (control)]. **(A)** The number of differential metabolites increased or decreased between the positive and control groups for different antibodies. “up” represents the number of metabolites enriched in the antibody positive group, while “down” represents the number of metabolites enriched in the control group. **(B)** Bubble charts representing the characteristic metabolic pathways of three antibody positive groups. **(C)** Differential metabolites Venn diagrams among three groups of DM involving different antibody positive. **(D)** ROC curves for distinguishing MDA5+DM group based on combined metabolites panel of ST 24:2;O3;S and sphinganine-1-phosphate. **(E)** ROC curves for distinguishing TIF1-γ+DM group based on combined metabolites panel of pro-leu, glutamine, and sphingosine-1-phosphate. **(F)** ROC curves for distinguishing Jo-1+DM group based on combined metabolites panel of CPA (18:0/0:0), glutamic acid, and LPC O-17:1. * p<0.05, ** p<0.01, *** p<0.001, **** p<0.0001.

Using VIP>1, *p*<0.05, and FC>1.2 or FC<0.8 as screening conditions, the differential metabolites between the MDA5+DM, TIF1-γ+DM, and Jo-1+DM groups and control were identified (**Supplementary Figure S11**). A total of 26 differential metabolites between the MDA5+DM and control groups were identified, of which 13 metabolites were enriched in the MDA5+DM group; 20 different metabolites between TIF1-γ+DM group and control group were found, of which 10 metabolites were enriched in TIF1-γ+DM group; and 23 differential metabolites between the Jo-1+DM and control groups were screened, and only eight metabolites were enriched in Jo-1+DM (**Figure 4A**). Furthermore, the metabolic pathways involved in the differential metabolites of each group were analyzed by MetaboAnalyst 5.0. As shown in **Figure 4B**, the most important metabolic pathways for the MDA5+DM were retinol, sphingolipid metabolism, citrate cycle, glycerophopholipid metabolism, and purine metabolism, differencing from TIF1-γ+DM and Jo-1+DM, whose differential metabolic pathways involved D-glutamine and D-glutamate metabolism; alanine, aspartate, and glutamate metabolism; and arginine biosynthesis.

The differential metabolites between DM with different antibody (MDA5+DM, TIF1-γ+DM, and Jo-1+DM) and control groups were made into Venn maps to obtain unique biomarkers of different antibody positive DM (**Figure 4C**). There were 18, 12, or 18 characteristic metabolites in MDA5+, TIF1-γ+, or Jo-1+DM groups, respectively. Univariate ROC analysis revealed lower AUC for these specific metabolites in different groups. Furthermore, stepwise forward logistic regression was used to select altered metabolites in each group. The panel composed of ST 24:2;O3;S and sphinganine 1-phosphate can distinguish the MDA5+DM group from the HC/control/TIF1-γ+/Jo-1+DM, with the AUC of 0.962 based on the combined ROC analysis (**Figure 4D**). A panel composed of pro-leu, glutamine, and sphingosine 1-phosphate can distinguish the TIF1-γ+DM group from the HC/control/MDA5+/Jo-1+DM, with the AUC of 0.928 (**Figure 4E**). A panel composed of CPA(18:0/0:0), glutamic acid, and LPC O-17:1 can distinguish the Jo-1+DM group from the HC/control/MDA5+/TIF1-γ+DM, with the AUC of 0.977 (**Figure 4F**).

## Discussion

**Figure 5:**
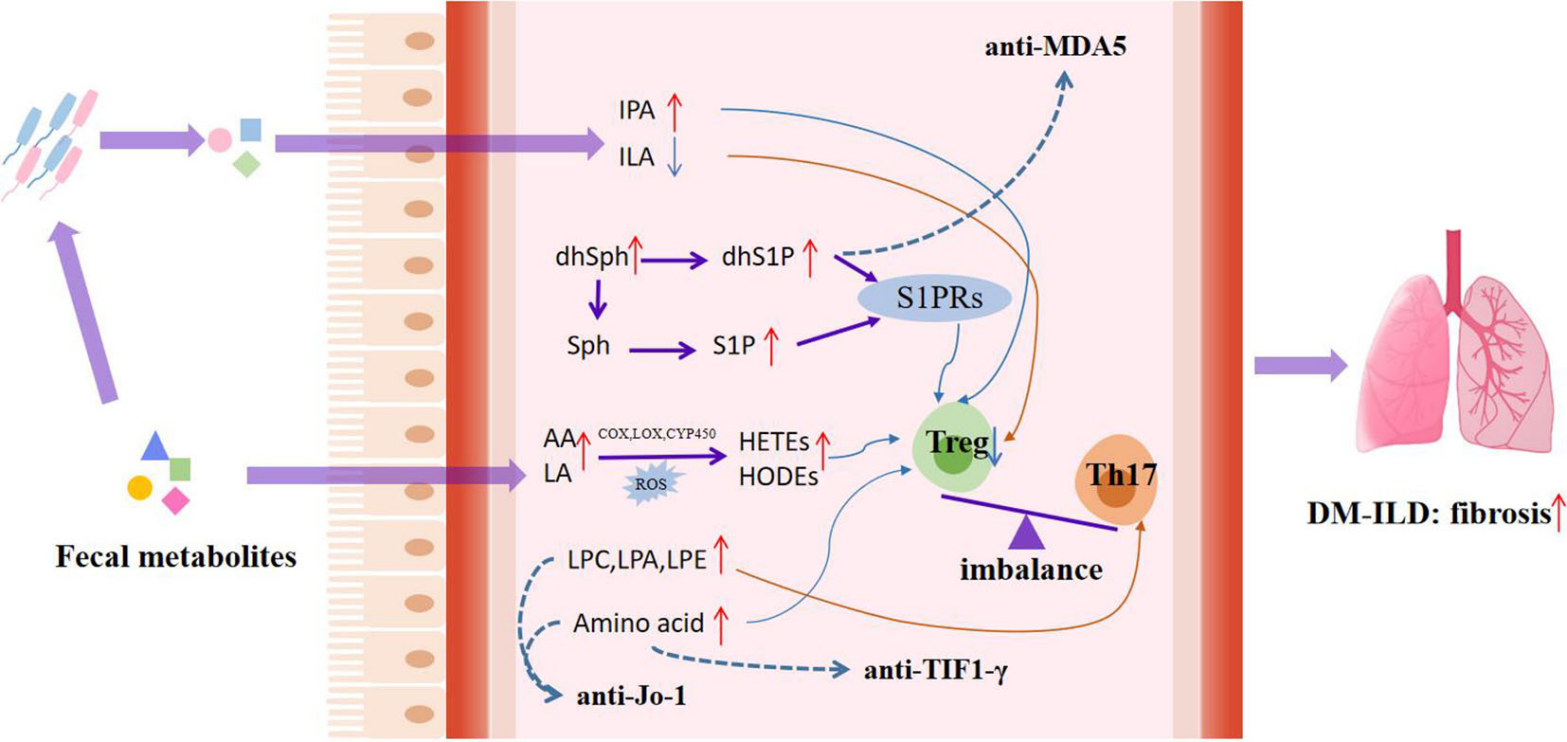
Potential mechanisms of dysregulated serum metabolites in pathogenesis of DM progression. Lipid abnormalities associated with DM, such as peroxidation of unsaturated fatty acids and abnormal metabolism of glycerolipid and sphingolipid, act on the immune system, thereby accelerating disease progression. The red short arrow indicates upregulation, and the blue short arrow indicates downregulation; the red long continuous line represents the promoting effect, and the blue long continuous line represents the inhibiting effect; The dashed blue line shows the effect of the metabolite on the myositis-specific autoantibodies expression. IPA, indolepropionic acid; ILA, indole-3-lactic acid; dhSph, dihydrosphingosine; dhS1P, dihydrosphingosine-1-phosphate; Sph, sphingosine; S1P, Sphingosine-1-phosphate; AA, arachidonic acid; LA, linoleic acid; S1PRs, G-protein-coupled S1P receptors; HODEs, hydroxy-octadecene dienoic acid; HETEs, hydroxyeicosatetraenoic acid; LPC, lysophosphatidylcholine; LPE, lysophosphatidylethanolamine; LPA, lysophosphatidic acid.

In this study, we detected the serum metabolic profile of DM based on high-resolution mass spectrometry combined with machine learning methods to determine the biomarkers of DM. Meanwhile, altered metabolic profiles of DM with different MSAs associated with the disease activity and interstitial lung disease were studied for the first time, which also helps to understand the molecular mechanism of the development of DM. Based on above findings, we summarized the metabolic characteristics of different types of DM and proposed the potential mechanism of dysregulation of serum metabolites in the pathogenesis of DM progression, as shown in **Table 2** and **Figure 5**.

**Table 2:** Metabolic, immune and clinical characteristics of different types of DM.

DM classification	Altered metabolites	Pathways	Immune characteristic	Clinical phenotype
H-DM	Pro-leu, 9-OxoODE, 2-hydroxybutyric acid, FA 14:0;O, FA 20:5;O2 and 11-HpODE, creatine↑	Starch and sucrose metabolism; Propanoate metabolism; Amino acid metabolism; Aminoacyl-tRNA biosynthesis	Treg↓	Skin lesions; muscle weakness; hoarseness or sore throat, dysphagia; arthralgia;↑ High antibody positive rate
DM-ILD	ILA, dehydroepiandrosterone↓ Sph, dhSph, dhS1P, cholic Acid, SM 32:1;O2↑	Sphingolipid metabolism; Androgen and estrogen Metabolismitryptophan metabolism	Treg↓ Th17/Treg↑ (16)	High antibody positive ratet cardiac involvementd Gottron’s papules or sign↑
MDA5+DM	S1P, dhS1P, ST 24:1, ST 24:2↑; LTB5, 5-HETE, 4-HDoHE, 15S-HETrE, Succinic acid↓	Sphingolipid metabolism; Citrate cycle; arachidonic acid metabolism	T cell↓, Th1↓	Skin lesions; ILD; Cardiac involvement ↑ (17)
TIF1-γ+DM	Pro-leu, carnitine, glutamine, S1P, dhS1P↑; 9-HOTrE, LTB5, glutamic acid, 12(S)-HETrE, succinic acid↓	Sphingolipid metabolism; Amino acid metabolism	T cell↓	Muscle weakness; heliotrope rash; malignancies (18, 19)
Jo-1+DM	Glutamic acid↑; LPA 18:0, LPC 18:0, LPC 18:2, LPC 16:0, LPC 22:4, LPE 18:2, LPE 20:3, CPA(18:0/0:0)↓	Amino acid metabolism; Glycerophospholipid metabolism	NK↑, Th1↑, Treg↓	CK↑, Arthralgia↑, Skin lesions↓ (8)

An upward arrow indicates an increase, and a downward arrow indicates a decrease. H-DM, high disease activity DM; ILD, interstitial lung disease; DM-ILD, DM-associated with ILD; FA, fatty acyls; ILA, indole-3-lactic acid; Sph, sphingosine; dhSph, dihydrosphingosine; dhS1P, dihydrosphingosine-1-phosphate; SM, sphingomyelin; ST, sterols; LTB5, leukotriene B5; LPA, lysophosphatidic acid; LPC, lysophosphatidylcholine; LPE, lysophosphatidylethanolamine; CPA, cyclic phosphatidic acid; CK, creatine kinase.

Previous studies have found abnormal lipid metabolism in DM (14, 15, 20). In this study, serum metabolic profiles can distinguish DM from HCs. Among these, the changes in the lipid metabolism of DM were the most obvious, especially glycerophospholipids and unsaturated fatty acids. Here, we found an increase in lysophospholipids, including lysophosphatidylcholine (LPC), lysophosphatidylethanolamine (LPE), lysophosphatidic acid (LPA), lysophosphatidylinositol (LPI), and phosphatidylcholine (PC) in DM serum.

LPC is one of the most abundant lipids in blood and has been reported to have inflammatory, anti-hemostatic, and cytotoxic effects (21). In certain contexts, LPC, such as saturated (16:0 and 18:0) and monounsaturated (18:1) LPC, have been shown to have pro-inflammatory effects through the upregulation of adhesion molecules, increased release of chemokines, and production of reactive oxygen species (ROS) (22, 23). Previous studies have shown that LPC was elevated in autoimmune-related diseases, including myositis, psoriasis, and lupus (23). LPC plays a role through different signaling pathways such as NF-κB, PKC, and ERK signaling in multiple cell types (such as T lymphocytes, monocytes, and neutrophils) (24). Besides LPC, LPA, LPE, and LPI play essential roles in various cellular and inflammatory responses (23, 24). For example, LPA initiates signaling pathways or exerts biological effects through different receptor subtypes, promotes cell growth and differentiation through LPA receptors 1, 3, and PPAR-γ, and contributes to mast cell proliferation during inflammation. LPI activates signaling pathways related to cell proliferation, migration, and tumorigenesis and plays a vital role as an active lipid mediator and initiates multiple mechanisms through its interaction with G-protein-coupled receptor 55 (GPR55) and Ca^2+^ ion channel mechanisms cell response (24, 25).

In addition, our results showed that unsaturated fatty acids, especially linoleic acid (LA), arachidonic acid (AA), and their oxidized derivatives, were elevated in DM serum. AA promoted in DM has been shown to be converted into various metabolites by cyclooxygenase (COX), lipoxygenase (LOX), and cytochrome P450 (CYP450) enzymes, such as prostaglandins and leukotrienes, triggering different inflammatory responses (26, 27). Although the effects of LA and its derivatives on inflammation were less studied, recent studies have demonstrated that its oxidative metabolites contributed to inflammatory pain in essence (28). LA is an essential fatty acid that can be oxidized by endogenous enzymes and reactive oxygen species in the circulation to synthesize a series of oxidative derivatives that play key roles in regulating inflammation (29). For example, LA is metabolized by LOX into hydroxy-octadecene dienoic acid (HODEs) derivatives (such as 9- and 13-HODE) and is further converted into oxygen-HODE (such as 9-oxygen-HODE and 13-oxygen-HODE) and epoxy-HODE, which play a role in inflammation (30). These important pro-inflammatory biomarker factors, such as 9-HODE and 13-HODE, were elevated in DM in our study.

The generation of a large amount of oxidized lipids might indicate an imbalance between oxidation and antioxidant activity in DM. The reactive oxygen species produced by oxidative stress state can disrupt different signaling pathways, which are related to the loss of regulation of immune inflammatory response, especially the response of Treg (31). Previous literature has shown that oxidative stress is a key factor in the progression and deterioration of autoimmune diseases, possibly by further inducing and expanding the expansion of pro-inflammatory Th17 cells, inhibiting the differentiation of anti-inflammatory Treg cells, and exacerbating autoimmune damage (32–34). In summary, lipid metabolism dysfunction is one of the characteristics of DM, which is consistent with previous studies (35).

The altered metabolic profile was also associated with disease activity. 2-Hydroxybutyric acid (2-HB) was found to have a strong correlation with disease activity, with higher levels in H-DM. 2-HB, which is mainly produced during L-threonine metabolism or glutathione synthesis, is an important regulator, and its underlying biochemical mechanisms might be involved in lipid oxidation and increased oxidative stress (36). Low levels of creatinine and high levels of creatine in the serum of DM patients were also associated with myositis damage. Under stable kidney function, the concentration of serum creatinine can reflect skeletal muscle mass (37). When muscle damage occurs, it can impair the muscle’s ability to uptake or retain creatine. This impairment leads to an increased release of creatine, which is predominantly stored in the muscle tissue, into the serum. Consequently, this results in elevated serum creatine levels and a decrease in muscle creatine concentration. This reduction in muscle creatine availability subsequently leads to a decrease in creatinine production (38). Moreover, studies had shown that creatine may affect cytokines through the NF-κB signaling pathway, thereby affecting cytokines, receptors, or growth factors, and thus having a positive or negative impact on the immune response (39).

To be emphasized, differential metabolites were also found between DM-ILD and DM-nonILD in this study, mainly including glycerolipid, sphingomyelin, and LPE. Lipid dysregulation has been well described in several lung diseases, including cystic fibrosis, asthma, and chronic obstructive pulmonary disease (COPD) (40). A study on the serum metabolomic profile of ILD in RA patients showed that glycerol was higher in serum in RA-ILD relative to RA without ILD (41). In this study, a total of five differential metabolites, including indole-3-lactic acid, dihydrosphingosine, SM 32:1;O2, NAE 17:1, and cholic acid, were screened as potential biomarkers to distinguish DM-ILD from DM-nonILD. Indole-3-lactic acid (ILA) is an indole derivative that is involved in the metabolism of tryptophan by gut microbiota. Indole derivatives participate in the differentiation of immune cells and the synthesis of cytokines through aromatic hydrocarbon receptors, to regulate immunity and participate in anti-inflammatory and allergic reactions (42–45). It may directly or indirectly participate in the immune regulation of lung disease hosts by stimulating the distal immune response through the “gut–lung axis” (46–48). In addition, research had reported that dihydrosphingosine (dhSph) was significantly elevated in the stratum corneum of the lesional skin in atopic dermatitis and was associated with skin barrier function, disease severity, and local cytokine levels (49). In addition, dhSph metabolism forms dihydrosphingosine-1-phosphate (dhS1P) under the action of sphingosine kinase, which can increase collagen synthesis in fibroblasts leading to fibrosis through dhS1P JAK/STAT-TIMP1 signaling (50, 51).

Interestingly, in order to investigate whether metabolic changes are related to the expression of myositis specific antibodies, we analyzed the serum metabolic profiles of different antibody-positive DM and found that DM with different specific antibody expressions had unique metabolic characteristics. For the MDA5+DM group, we found that a panel composed of ST 24:2;O3;S and sphinganine-1-phosphate (dhS1P) can distinguish the MDA5+DM group from the HC/control/TIF1-γ+/Jo-1+DM. Both dhS1P and its analogue sphingosine-1-phosphate (S1P) have been found to bind to the G-protein-coupled S1P receptor (S1PR1) to regulate immune responses (52, 53). Phosphorylation and subsequent internalization of S1PR1 in T cells modulates the polarization of Th17, thereby inducing a pro-inflammatory immune response. In addition, dhS1P and S1P can induce fibroblasts to produce collagen and promote the formation of fibrosis (51, 54). The expression levels of SIP and dhS1P were higher in MDA5+DM group than in other groups, and the expression levels of dhSph (the precursor of dhS1P) were increased in DM-ILD, suggesting a potential relationship between MDA5+ and ILD, and the important role of sphingosine metabolism in anti-MDA5+ and ILD. Additionally, MDA5+DM patients were prone to respiratory failure; the dysregulation of serum lipid metabolism and citrate cycle in MDA5+DM might be related to hypoxia response. The hypoxic environment could lead to increased accumulation of reactive oxygen species (ROS) and oxidative stress, which induces the activation of HIF transcription factors and regulates lipid metabolism through multiple pathways (55–58). In addition, the upregulation of hypoxia response is associated with an increase in the infiltration of activated inflammatory cells, accompanied by an increase in metabolic demand (59).

In addition, among the characteristic metabolites of TIF1-γ+DM, the metabolic pathways involved were mainly amino acid metabolism, including D-glutamine and D-glutamate metabolism; alanine, aspartate, and glutamate metabolism; and arginine synthesis. Glutamine, serine, and glycine have been identified to function as metabolic regulators in supporting cancer cell growth (60). In the Jo-1+DM, there were certain changes in amino acid metabolism and glycerol phospholipid metabolism. However, further functional metabolic research is needed to investigate how these metabolites affect antibody expression and subsequently affect clinical pathological changes.

The limitations of this study include the small sample size and the lack of accurate quantitative verification in the external cohort. Although we have found the characteristic metabolites in the serum of simple MDA5+DM, Jo-1+DM, and TIF1-γ+DM, there were fewer samples with simple positive antibodies in our study, and the results obtained need to be verified in the cohorts with a larger sample size.

In summary, this study identified altered metabolic profiles of dermatomyositis with different myositis-specific autoantibodies, which may be associated with the disease activity and interstitial lung disease. Metabolic biomarkers of different classifications of DM that can monitor disease activity, predict patient prognosis, help early diagnosis, and/or select therapeutic targets for DM. At the same time, the unique metabolic profile of each antibody-positive DM helps to explore the affected signaling pathway in the occurrence and development of DM.

## Data Availability

The raw data supporting the conclusions of this article will be made available by the authors, without undue reservation.

## Glossary

2-HB: 2-hydroxybutyric acid
AA: arachidonic acid
AUC: area under the curve
COPD: chronic obstructive pulmonary disease
COX: cyclooxygenase
CYP450: cytochrome P450
dhS1P: dihydrosphingosine-1-phosphate/sphinganine-1-phosphate
dhSph: dihydrosphingosine
DM: Dermatomyositis
DM-ILD: DM-associated with ILD
DM-nonILD: DM without ILD
FC: fold change
FDR: false discovery rate
GPR55: G protein-coupled receptor 55
HCs: healthy controls
H-DM: high disease activity DM
HODEs: hydroxy-octadecene dienoic acid
ILA: Indole-3-lactic acid
ILD: interstitial lung disease
LA: linoleic acid
L-DM: low disease activity DM
LOX: lipoxygenase
LPA: lysophosphatidic acid
LPC: lysophosphatidylcholine
LPE: lysophosphatidylethanolamine
LPI: lysophosphatidylinositol
MSAs: myositis-specific autoantibodies
MYOACT: Myositis Disease Activity Assessment Visual Analogue Scales
OPLS-DA: orthogonal partial least square discriminant analysis
PC: phosphatidylcholine
PCA: principal component analysis
PLS-DA: partial least squares discrimination analysis
QC: quality control
RF: random forest
ROC: Receiver operating characteristic
ROS: reactive oxygen species
S1P: sphingosine-1-phosphate
S1PR1: G-protein-coupled S1P receptor
SVM: support vector machines
UPLC-TOF-MS: ultra-high performance liquid chromatography-composed time-of-flight mass spectrometry
VIP: variable importance in the projection

## References

[1] DeWane ME Waldman R Lu J. Dermatomyositis: Clinical features and pathogenesis. J Am Acad Dermatol. (2020) 82:267–81. doi: 10.1016/j.jaad.2019.06.1309 31279808

[2] McPherson M Economidou S Liampas A Zis P Parperis K. Management of MDA-5 antibody positive clinically amyopathic dermatomyositis associated interstitial lung disease: A systematic review. Semin Arthritis Rheum. (2022) 53:151959. doi: 10.1016/j.semarthrit.2022.151959 35134633

[3] Fujisawa T. Management of myositis-associated interstitial lung disease. Medicina. (2021) 57:347. doi: 10.3390/medicina57040347 33916864 PMC8065549

[4] Sun KY Fan Y Wang YX Zhong YJ Wang GF. Prevalence of interstitial lung disease in polymyositis and dermatomyositis: A meta-analysis from 2000 to 2020. Semin Arthritis Rheum. (2021) 51:175–91. doi: 10.1016/j.semarthrit.2020.11.009 33383294

[5] Xu L You H Wang L Lv C Yuan F Li J. Identification of three different phenotypes in anti-MDA5 antibody-positive dermatomyositis patients: implications for rapidly progressive interstitial lung disease prediction. Arthritis Rheumatol. (2022) 75:609–19. doi: 10.1002/art.42308 35849805

[6] Sevim E Kobrin D Casal-Dominguez M Pinal-Fernandez I. A comprehensive review of dermatomyositis treatments - from rediscovered classics to promising horizons. Expert Rev Clin Immunol. (2023) 16:1–13. doi: 10.1080/1744666X.2023.2270737 PMC11611049 37842905

[7] McHugh NJ Tansley SL. Autoantibodies in myositis. Nat Rev Rheumatol. (2018) 14:290–302. doi: 10.1038/nrrheum.2018.56 29674612

[8] de Andrade VP De Souza FHC Behrens Pinto GL Shinjo SK. The relevance of anti-Jo-1 autoantibodies in patients with definite dermatomyositis. Adv Rheumatol. (2021) 61:12. doi: 10.1186/s42358-021-00171-x 33608062

[9] Bolko L Gitiaux C Allenbach Y. Dermatomyositis: new antibody, new classification. Med Sci (Paris). (2019) 35 Hors série n° 2:18–23. doi: 10.1051/medsci/2019178 31859626

[10] Wu W Guo L Fu Y Wang K Zhang D Xu W. Interstitial lung disease in anti-MDA5 positive dermatomyositis. Clin Rev Allergy Immunol. (2021) 60:293–304. doi: 10.1007/s12016-020-08822-5 33405101

[11] Castillo RL Femia AN. Polishing the crystal ball: mining multi-omics data in dermatomyositis. Ann Transl Med. (2021) 9:435. doi: 10.21037/atm-20-5319 33842656 PMC8033302

[12] Gao S Luo H Zhang H Zuo X Wang L Zhu H. Using multi-omics methods to understand dermatomyositis/polymyositis. Autoimmun Rev. (2017) 16:1044–8. doi: 10.1016/j.autrev.2017.07.021 28778709

[13] Zhang T Xu J Liu Y Liu J. Metabolomic profiling for identification of potential biomarkers in patients with dermatomyositis. Metabolomics. (2019) 15:77. doi: 10.1007/s11306-019-1539-9 31087209

[14] Raouf J Idborg H Englund P Alexanderson H Dastmalchi M Jakobsson PJ. Targeted lipidomics analysis identified altered serum lipid profiles in patients with polymyositis and dermatomyositis. Arthritis Res Ther. (2018) 20:83. doi: 10.1186/s13075-018-1579-y 29720222 PMC5932839

[15] Dvergsten JA Reed AM Landerman L Pisetsky DS Ilkayeva O Huffman KM. Metabolomics analysis identifies a lipidomic profile in treatment-naïve juvenile dermatomyositis patients vs healthy control subjects. Rheumatology. (2022) 61:1699–708. doi: 10.1093/rheumatology/keab520 PMC8996785 34185053

[16] Feng M Guo H Zhang C Wang Y Liang Z Zhao X. Absolute reduction of regulatory T cells and regulatory effect of short-term and low-dose IL-2 in polymyositis or dermatomyositis. Int Immunopharmacol. (2019) 77:105912. doi: 10.1016/j.intimp.2019.105912 31669890

[17] Nombel A Fabien N and Coutant F. Dermatomyositis with anti-MDA5 antibodies: Bioclinical features, pathogenesis and emerging therapies. Front Immunol. (2021) 12:773352. doi: 10.3389/fimmu.2021.773352 34745149 PMC8564476

[18] Kilinc OC Ugurlu S. Clinical features of dermatomyositis patients with anti-TIF1 antibodies: A case based comprehensive review. Autoimmun Rev. (2023) 22:103464. doi: 10.1016/j.autrev.2023.103464 37863375

[19] Ikeda N Yamaguchi Y Kanaoka M Ototake Y Akita A Watanabe T. Clinical significance of serum levels of anti-transcriptional intermediary factor 1-γ antibody in patients with dermatomyositis. J Dermatol. (2020) 47:490–6. doi: 10.1111/1346-8138.15284 32103537

[20] Huang W Ren F Luo L Zhou J Huang D Tang L. Clinical characteristics of lipid metabolism in untreated patients with anti-MDA5 antibody-positive. Int J Gen Med. (2021) 14:2507–12. doi: 10.2147/IJGM.S315885 PMC8214207 34163218

[21] Tan ST Ramesh T Toh XR Nguyen LN. Emerging roles of lysophospholipids in health and disease. Prog Lipid Res. (2020) 80:101068. doi: 10.1016/j.plipres.2020.101068 33068601

[22] Zeng C Wen B Hou G Lei L Mei Z Jia X. Lipidomics profiling reveals the role of glycerophospholipid metabolism in psoriasis. GigaScience. (2017) 6:1–11. doi: 10.1093/gigascience/gix087 PMC5647792 29046044

[23] Zhang C Wang K Yang L Liu R Chu Y Qin X. Lipid metabolism in inflammation-related diseases. Analyst. (2018) 143:4526–36. doi: 10.1039/C8AN01046C 30128447

[24] O'Donnell VB Rossjohn J Wakelam MJ. Phospholipid signaling in innate immune cells. J Clin Invest. (2018) 128:2670–9. doi: 10.1172/JCI97944 PMC6026006 29683435

[25] Desale SE Chinnathambi S. Phosphoinositides signaling modulates microglial actin remodeling and phagocytosis in Alzheimer’s disease. Cell Commun Signal. (2021) 19:28. doi: 10.1186/s12964-021-00715-0 33627135 PMC7905611

[26] Korotkova M Lundberg IE. The skeletal muscle arachidonic acid cascade in health and inflammatory disease. Nat Rev Rheumatol. (2014) 10:295–303. doi: 10.1038/nrrheum.2014.2 24468934

[27] Wang T Fu X Chen Q Patra JK Wang D Wang Z. Arachidonic acid metabolism and kidney inflammation. Int J Mol Sci. (2019) 20:3683. doi: 10.3390/ijms20153683 31357612 PMC6695795

[28] Wedel S Osthues T Zimmer B Angioni C Geisslinger G Sisignano M. Oxidized linoleic acid metabolites maintain mechanical and thermal hypersensitivity during sub-chronic inflammatory pain. Biochem Pharmacol. (2022) 198:114953. doi: 10.1016/j.bcp.2022.114953 35149052

[29] Innes JK Calder PC. Omega-6 fatty acids and inflammation. Prostaglandins Leukot Essent Fatty Acids. (2018) 132:41–8. doi: 10.1016/j.plefa.2018.03.004 29610056

[30] Hildreth K Kodani SD Hammock BD Zhao L. Cytochrome P450-derived linoleic acid metabolites EpOMEs and DiHOMEs: a review of recent studies. J Nutr Biochem. (2020) 86:108484. doi: 10.1016/j.jnutbio.2020.108484 32827665 PMC7606796

[31] Lightfoot YL Blanco LP Kaplan MJ. Metabolic abnormalities and oxidative stress in lupus. Curr Opin Rheumatol. (2017) 29:442–9. doi: 10.1097/BOR.0000000000000413 PMC5586499 28639951

[32] Alissafi T Kalafati L Lazari M Filia A Kloukina I Manifava M. Mitochondrial oxidative damage underlies regulatory T cell defects in autoimmunity. Cell Metab. (2020) 32:591–604.e7. doi: 10.1016/j.cmet.2020.07.001 32738205 PMC7611060

[33] Chávez MD Tse HM. Targeting mitochondrial-derived reactive oxygen species in T cell-mediated autoimmune diseases. Front Immunol. (2021) 12:703972. doi: 10.3389/fimmu.2021.703972 34276700 PMC8281042

[34] Yang J Yang X Zou H Li M. Oxidative stress and Treg and Th17 dysfunction in systemic lupus erythematosus. Oxid Med Cell Longev. (2016) 2016:2526174. doi: 10.1155/2016/2526174 27597882 PMC4997077

[35] Vasiljevski ER Summers MA Little DG Schindeler A. Lipid storage myopathies: Current treatments and future directions. Prog Lipid Res. (2018) 72:1–17. doi: 10.1016/j.plipres.2018.08.001 30099045

[36] Qin F Li J Mao T Feng S Li J Lai M. 2 hydroxybutyric acid-producing bacteria in gut microbiome and Fusobacterium nucleatum regulates 2 hydroxybutyric acid level *in vivo* . Metabolites. (2023) 13:451. doi: 10.3390/metabo13030451 36984891 PMC10059959

[37] Patel SS Molnar MZ Tayek JA Ix JH Noori N Benner D. Serum creatinine as a marker of muscle mass in chronic kidney disease: results of a cross-sectional study and review of literature. J Cachexia Sarcopenia Muscle. (2013) 4:19–29. doi: 10.1007/s13539-012-0079-1 22777757 PMC3581614

[38] Wyss M Kaddurah-Daouk R. Creatine and creatinine metabolism. Physiol Rev. (2000) 80:1107–213. doi: 10.1152/physrev.2000.80.3.1107 10893433

[39] Kreider RB Stout JR. Creatine in health and disease. Nutrients. (2021) 13:447. doi: 10.3390/nu13020447 33572884 PMC7910963

[40] Nambiar S Bong How S Gummer J Trengove R Moodley Y. Metabolomics in chronic lung diseases. Respirology. (2020) 25:139–48. doi: 10.1111/resp.13530 30907495

[41] Furukawa H Oka S Shimada K Hashimoto A Komiya A Matsui T. Serum metabolomic profiles of rheumatoid arthritis patients with acute-onset diffuse interstitial lung disease. biomark Insights. (2019) 14:1177271919870472. doi: 10.1177/1177271919870472 31488947 PMC6709435

[42] Ehrlich AM Pacheco AR Henrick BM Taft D Xu G Huda MN. Indole-3-lactic acid associated with Bifidobacterium-dominated microbiota significantly decreases inflammation in intestinal epithelial cells. BMC Microbiol. (2020) 20:357. doi: 10.1186/s12866-020-02023-y 33225894 PMC7681996

[43] Meng D Sommella E Salviati E Campiglia P Ganguli K Djebali K. Indole-3-lactic acid, a metabolite of tryptophan, secreted by Bifidobacterium longum subspecies infantis is anti-inflammatory in the immature intestine. Pediatr Res. (2020) 88:209–17. doi: 10.1038/s41390-019-0740-x PMC7363505 31945773

[44] Kim K Kim H Sung GY. Effects of indole-3-lactic acid, a metabolite of tryptophan, on IL-4 and IL-13-induced human skin-equivalent atopic dermatitis models. Int J Mol Sci. (2022) 23:13520. doi: 10.3390/ijms232113520 36362303 PMC9655012

[45] Zhang FL Chen XW Wang YF Hu Z Zhang WJ Zhou BW. Microbiota-derived tryptophan metabolites indole-3-lactic acid is associated with intestinal ischemia/reperfusion injury via positive regulation of YAP and Nrf2. J Transl Med. (2023) 21:264. doi: 10.1186/s12967-023-04109-3 37072757 PMC10111656

[46] Dang AT Marsland BJ. Microbes, metabolites, and the gut-lung axis. Mucosal Immunol. (2019) 12:843–50. doi: 10.1038/s41385-019-0160-6 30976087

[47] Gong G Song S Su J. Pulmonary fibrosis alters gut microbiota and associated metabolites in mice: An integrated 16S and metabolomics analysis. Life Sci. (2021) 264:118616. doi: 10.1016/j.lfs.2020.118616 33098825

[48] Ma PJ Wang MM Wang Y. Gut microbiota: A new insight into lung diseases. BioMed Pharmacother. (2022) 155:113810. doi: 10.1016/j.biopha.2022.113810 36271581

[49] Toncic RJ Jakasa I Hadzavdic SL Goorden SM Vlugt KJG Stet FS. Altered levels of sphingosine, sphinganine and their ceramides in atopic dermatitis are related to skin barrier function, disease severity and local cytokine Milieu. Int J Mol Sci. (2020) 21:1958. doi: 10.3390/ijms21061958 32183011 PMC7139865

[50] Magaye RR Savira F Hua Y Kelly DJ Reid C Flynn B. The role of dihydrosphingolipids in disease. Cell Mol Life Sci. (2019) 76:1107–34. doi: 10.1007/s00018-018-2984-8 PMC11105797 30523364

[51] Magaye RR Savira F Hua Y Xiong X Huang L Reid C. Exogenous dihydrosphingosine 1 phosphate mediates collagen synthesis in cardiac fibroblasts through JAK/STAT signalling and regulation of TIMP1. Cell Signal. (2020) 72:109629. doi: 10.1016/j.cellsig.2020.109629 32278008

[52] Ranjit DK Moye ZD Rocha FG Ottenberg G Nichols FC Kim HM. Characterization of a bacterial kinase that phosphorylates dihydrosphingosine to form dhS1P. Microbiol Spectr. (2022) 10:e0000222. doi: 10.1128/spectrum.00002-22 35286133 PMC9045371

[53] Matwiejuk M Mysliwiec H Lukaszuk B Lewoc M Malla H Mysliwiec P. The interplay between bioactive sphingolipids in the psoriatic skin and the severity of the disease. Int J Mol Sci. (2023) 24:11336. doi: 10.3390/ijms241411336 37511095 PMC10378918

[54] Magaye RR Savira F Hua Y Xiong X Huang L Reid C. Attenuating PI3K/Akt- mTOR pathway reduces dihydrosphingosine 1 phosphate mediated collagen synthesis and hypertrophy in primary cardiac cells. Int J Biochem Cell Biol. (2021) 134:105952. doi: 10.1016/j.biocel.2021.105952 33609744

[55] Lee P Chandel NS Simon MC. Cellular adaptation to hypoxia through hypoxia inducible factors and beyond. Nat Rev Mol Cell Bio. (2020) 21:268–83. doi: 10.1038/s41580-020-0227-y PMC7222024 32144406

[56] Krishnan J Suter M Windak R Krebs T Felley A Montessuit C. Activation of a HIF1α-PPARγ axis underlies the integration of glycolytic and lipid anabolic pathways in pathologic cardiac hypertrophy. Cell Metab. (2009) 9:512–24. doi: 10.1016/j.cmet.2009.05.005 19490906

[57] Du W Zhang L Brett-Morris A Aguila B Kerner J Hoppel CL. HIF drives lipid deposition and cancer in ccRCC via repression of fatty acid metabolism. Nat Commun. (2017) 8:1769. doi: 10.1038/s41467-017-01965-8 29176561 PMC5701259

[58] Seo J Jeong DW Park JW Lee KW Fukuda J Chun YS. Fatty-acid-induced FABP5/HIF-1 reprograms lipid metabolism and enhances the proliferation of liver cancer cells. Commun Biol. (2020) 3:638. doi: 10.1038/s42003-020-01367-5 33128030 PMC7599230

[59] Kellett SK Masterson JC. Cellular metabolism and hypoxia interfacing with allergic diseases. J Leukoc Biol. (2024) 116:335–48. doi: 10.1093/jleuko/qiae126 38843075

[60] Li Z Zhang H. Reprogramming of glucose, fatty acid and amino acid metabolism for cancer progression. Cell Mol Life Sci. (2016) 73:377–92. doi: 10.1007/s00018-015-2070-4 PMC11108301 26499846

