# Supplementary material for: Altered metabolic profiles of dermatomyositis with different myositis-specific autoantibodies associated with clinical phenotype
Source: Front Immunol. 2024 Nov 25;15:1429010. doi: 10.3389/fimmu.2024.1429010 (PMC11625817; doi:10.3389/fimmu.2024.1429010)
Supplement: Supplementary file 1 [file DataSheet1.docx]

**Supplementary Methods: Clinical data collection of all participant and non-targeted metabolic data acquisition for serum samples**

**Clinical data collection**

Complete blood count, erythrocyte sedimentation rate (ESR), C-reactive protein (CRP) and biochemical parameters (including transaminase, lactate dehydrogenase (LDH), blood urea nitrogen (BUN), creatinine (Cr), creatine kinase (CK), CKMB, and hydroxybutyric dehydrogenase (HBDH) were tested routinely in the initial assessment of DM patients. The myositis autoantibody panels, including 16 MSAs (anti-TIF1-γ, anti-MDA5, anti-Jo-1, anti-OJ, anti-NXP2, anti-ZoA, anti-Mi-2, anti-SRP, anti-PL7, anti-PL12, anti-HMGCR, anti-YARS, anti-Ku, anti-EJ, anti-SAE1 and anti-SAE2 antibodies) and 16 myositis associated antibodies (anti-Ro52, anti-Ro60, anti-DFS70, anti-PM-Scl75, anti-M2, anti-P0, anti-La, anti-RNP-A, anti-RNP-C, anti-RNP-68, anti-CENP-B, anti-CENP-B, anti-Scl70, anti-Th/To, NOR90 and anti-Fibrillarin antibodies), were tested by Microblot-Array. Moreover, peripheral lymphocytes and CD4+ T cell subset were analyzed by monoclonal antibodies on a BD-FACS-CANTO II flow cytometer (Becton Dickinson, USA) during routine laboratory assessments.

**Serum sample preparation**

All the serum samples were thawed on ice before LC-MS analysis previously described (1). Briefly, 200 μL of each serum sample was mixed with 800 μL of methanol. All of the samples were shaken for 30 s and subjected to ultrasound for 10 min. Then the mixture was incubated at -20 ℃ for 2 h to facilitate protein precipitation. The mixtures were then centrifuged at 13,000 rpm at 4 ℃ for 15 min and 500 μL supernatants were collected, followed by vacuum drying and re-dissolved with 200 μL of methanol/water (1:1, v/v) for LC-MS analysis. Aliquots of samples were mixed for the preparation of quality control (QC) samples to evaluate the reproducibility of the LC-MS analysis.

**LC-MS analysis**

Briefly, the metabolic profiles of the serum samples were performed on a UPLC (ExionLCTM AD) coupled with Triple TOF 5600+ mass spectrometer (American, AB Sciex). Chromatographic separation was acquired on a Waters Acquity UPLC HSS T3 (1.8 μm, 2.1 × 100 mm). The column temperature was set at 40 ℃ and the injected volume was 5 µL. Date acquisition was performed in full scan mode both in the positive and negative ion modes, and also coupled with information-dependent acquisition (IDA) trigger product ion scan modes. The parameters of the MS acquisition of electron spray ionization (ESI) source were listed as follows: ion spray voltage, 5500 V in the positive mode and -4500 V in the negative ion mode; nebulizer gas of 55 psi; heater gas of 55 psi; curtain gas of 35 psi; decluttering potential of 60 V (positive) and -60V(negative); collision energy of 30 eV (positive) and -30 eV (negative); turbo spray temperature of 550 ℃; the full scan range of 100-1500 m/z and the ion scan range of 50-1250 m/z with high sensitivity.

The mobile phase A was ACN/H2O (1:9 v/v) with 0.1% formic acid and the mobile phase B was ACN/H2O (9:1 v/v) with 0.1% formic acid. The gradient elution program was optimized as follows: 0-4 min, 100% A; 4-6 min, 75%A and 25% B; 6-25min, 100% B; 25-29 min, 100% B; 29-31min, 100% A; 31-33 min, 100% A. The flow rate was set at 0.3 mL/min.

**Date analysis**

The raw data was imported to XCMS (version 3.6.3) for automatic data prepossessing including peak picking and retention time correction. Subsequently, the substances with detection rate less than 50% or relative standard deviation greater than 30% were filtered. Then the resulting data matrix were imported into SIMCA 14.0 software (Umetrics, Sweden) for multivariate data analysis, including principal component analysis (PCA) and orthogonal partial least square discriminant analysis (OPLS-DA). The variable importance in the projection (VIP) values from OPLS-DA models, fold change (FC), and *p* value or false discovery rate (FDR) correction were performed to screen the differential metabolites. The metabolites were identified by OSI/SMMS software (Dalian ChemData Solution Information Technology Co., Ltd, PR China), MSDIAL (version 5.1.221218) and other online databases, including Human Metabolome Database (http://www.hmdb.ca/), Lipidmaps (https://lipidmaps.org/) and LipidBlast (https://fiehnlab.ucdavis.edu/projects/lipidblast).

**References**

1. Zhang Q, Li X, Yin X, Wang H, Fu C, Wang H, et al. Metabolomic profiling reveals serum L-pyroglutamic acid as a potential diagnostic biomarker for systemic lupus erythematosus. *Rheumatology* (2021) 60(2):598-606.

**Supplementary Table S1** Lymphocyte subpopulation date of DM patients.

|  | Discovery set (n=96) | | Validation set(n=40) | |
| --- | --- | --- | --- | --- |
|  | DM (n=67) | HC | DM (n=28) | HC |
| **Lymphocyte subpopulation** | | | | |
| T cell (CD3+CD19-) (median/μL, IQR) | 690 (473, 1218) |  | 715 (462, 1179) |  |
| B cell (CD3-CD19+) (median/μL, IQR) | 161 (103, 303) |  | 158 (82, 305) |  |
| Th cell (CD3+CD4+) (median/μL, IQR) | 380 (222, 658) |  | 386 (265, 591) |  |
| CD8+T cell (CD3+CD8+)( median/μL, IQR) | 235 (138, 431) |  | 329 (157, 470) |  |
| NK cell (CD3-, CD56+) (median/μL, IQR) | 101 (54, 155) |  | 94 (73, 206) |  |
| **CD4+ T cell subsets** |  |  |  |  |
| Th1( median/μL, IQR) | 23.22 (11.81, 60.06) |  | 26.91 (16.54, 57.59) |  |
| %Th1 | 6 (3.11, 11.12) |  | 8.01 (4.35, 13.49) |  |
| Th2( median/μL, IQR) | 4.38 (2.29, 9.69) |  | 4.98 (3.46, 9.56) |  |
| %Th2 | 1.1 (0.8, 1.75) |  | 1.09 (0.91, 1.56) |  |
| Th17( median/μL, IQR) | 3.28 (1.72, 6.40) |  | 2.96 (1.8, 6.28) |  |
| %Th17 | 0.83 (0.51, 1.25) |  | 1.02 (0.57, 1.26) |  |
| Treg( median/μL, IQR) | 22.06 (12.56, 40.64) |  | 18.19 (12.23, 35.15) |  |
| %Treg | 4.73 (3.58, 7.0) |  | 4.79 (3.55, 6.63) |  |
| Th1/Th2 | 6 (2.98, 12.51) |  | 5.33 (4.02, 12.69) |  |
| Th17/Treg | 0.18 (0.08, 0.34) |  | 0.15 (0.12, 0.27) |  |

**Supplementary Table S2** Differential metabolites between HC and DM groups.

|  | Compounds | VIP | FDR(*p*) | FC  (DM/HC) |
| --- | --- | --- | --- | --- |
| C1 | Propylparaben | 2.39234 | 8.08045E-11 | 0.12 |
| C2 | CMPF | 7.19566 | 3.24838E-11 | 0.15 |
| C3 | Tetrahydroaldosterone-3-glucuronide | 2.57168 | 4.43842E-19 | 0.22 |
| C4 | Chaetoglobosin N | 2.50137 | 4.41097E-14 | 0.25 |
| C5 | Dehydroepiandrosterone | 1.23779 | 2.9064E-08 | 0.32 |
| C6 | 3-Indolepropionic acid | 1.01297 | 0.000147451 | 0.37 |
| C7 | 3-methyl-2-oxovaleric acid | 3.79331 | 4.45785E-07 | 0.37 |
| C8 | Dehydroepiandrosterone sulfate | 11.4483 | 1.57917E-06 | 0.39 |
| C9 | 3-methyl-2-oxovaleric acid | 3.03153 | 2.8378E-07 | 0.43 |
| C10 | 5a-Dihydrotestosterone sulfate | 7.3092 | 0.000372244 | 0.46 |
| C11 | Hippurate | 1.04907 | 0.041114063 | 0.47 |
| C12 | Shikimic acid | 3.17752 | 0.00201929 | 0.53 |
| C13 | Trifluoroacetic acid | 1.22414 | 0.001585462 | 0.58 |
| C14 | 4-[(2S)-2-hydroxy-3-methyl-3-[(2S,3R,4S,5S,6R)-3,4,5-trihydroxy-6-(hydroxymethyl)oxan-2-yl]oxybutoxy]furo[3,2-g]chromen-7-one | 1.90861 | 0.000237902 | 0.61 |
| C15 | L-Carnitine | 2.34116 | 6.82709E-13 | 0.61 |
| C16 | 3-hydroxy-1,2-dimethylpyridin-4(1H)-one | 1.04526 | 5.7259E-10 | 0.61 |
| C17 | Hypoxanthine | 1.09402 | 0.030970701 | 0.67 |
| C18 | LPC17:2 | 5.61294 | 0.01198 | 1.51 |
| C19 | Azelaic acid | 1.03368 | 0.000452444 | 1.50 |
| C20 | Isoleucine | 4.43208 | 1.60985E-10 | 1.52 |
| C21 | Palmitoleic acid | 3.12944 | 0.005360729 | 1.55 |
| C22 | 8(R)-HPODE | 1.04179 | 0.000885024 | 1.54 |
| C23 | Phosphoric acid | 2.03151 | 1.02032E-06 | 1.55 |
| C24 | Butyl dodecanoate | 1.73106 | 0.000305014 | 1.59 |
| C25 | Linoleic acid | 10.0995 | 2.53526E-05 | 1.61 |
| C26 | Docosahexanoic acid | 5.92652 | 0.000269126 | 1.66 |
| C27 | Lysine | 1.09008 | 1.31067E-07 | 1.67 |
| C28 | taurochenodeoxycholic acid | 1.64668 | 0.03890157 | 1.73 |
| C29 | 2-Hydroxybutyric acid | 1.96204 | 3.97267E-06 | 1.73 |
| C30 | Resolvin D5 | 1.60521 | 0.000597942 | 1.74 |
| C31 | LPE O-17:1 | 1.02163 | 0.000823499 | 1.76 |
| C32 | C10-LAS (TENTATIVE) | 1.19113 | 0.000146926 | 1.78 |
| C33 | LTB5 | 1.11869 | 0.0034501 | 1.80 |
| C34 | LPI 20:4 | 2.65113 | 0.004318005 | 1.80 |
| C35 | (5Z,8Z,11Z,14Z,17Z)-Eicosapentaenoic acid ethyl ester | 1.0035 | 6.37913E-05 | 1.83 |
| C36 | LPE 20:4 | 2.34589 | 0.01439013 | 1.86 |
| C37 | glycoursodeoxycholic acid | 3.55556 | 0.0478735 | 1.86 |
| C38 | Resolvin D3/FA 22:6,O3 | 1.9455 | 2.45426E-05 | 1.87 |
| C39 | NCGC00381425-01!8-hydroxy-8-(3-octyloxiran-2-yl)octanoic acid | 1.16048 | 0.000017664 | 1.91 |
| C40 | SM 32:1;O2 | 2.83388 | 0.000119647 | 1.92 |
| C41 | 10-HDoHE | 2.56893 | 0.000120118 | 1.99 |
| C42 | LPE O-16:1 | 3.39462 | 1.54865E-06 | 2.03 |
| C43 | SM 34:1;O2 | 13.8144 | 3.66731E-06 | 2.05 |
| C44 | LPC 18:1 | 8.71788 | 0.00167112 | 2.045 |
| C45 | hexadecanoic acid/FA 16:0 | 8.12814 | 0.000197226 | 2.06 |
| C46 | FA 20:5;O2 | 1.01449 | 7.88284E-05 | 2.07 |
| C47 | Glycochenodexycholate | 1.31509 | 0.016682632 | 2.07 |
| C48 | PC 36:5 | 1.93091 | 3.97267E-06 | 2.08 |
| C49 | LPE O-18:2 | 2.5197 | 4.46159E-07 | 2.13 |
| C50 | Pro-Leu | 1.3473 | 3.45033E-07 | 2.15 |
| C51 | Choline | 5.51785 | 6.11595E-19 | 2.14 |
| C52 | LPE O-18:1 | 3.29696 | 2.2667E-05 | 2.18 |
| C53 | FA 18:1+1O | 1.33004 | 0.006769976 | 2.20 |
| C54 | LPC 17:1 | 6.38126 | 0.000621777 | 2.24 |
| C55 | Sphinganine 1-phosphate\|Dihydrosphingosine 1-phosphate | 1.8496 | 7.34495E-05 | 2.29 |
| C56 | 5(S),6(R),15(R)-Lipoxin A4 | 1.7619 | 6.87388E-08 | 2.32 |
| C57 | (Z)-5,8,11-trihydroxyoctadec-9-enoic acid | 2.48225 | 0.000397414 | 2.33 |
| C58 | PC 36:6 | 1.20406 | 8.1307E-08 | 2.34 |
| C59 | Linoleamide | 1.15069 | 0.005602043 | 2.35 |
| C60 | FA 20:2/Cis-11,14-Eicosadienoic acid | 1.15312 | 3.95141E-07 | 2.36 |
| C61 | FA 18:2+2O/11-HpODE | 2.88763 | 0.000151011 | 2.40 |
| C62 | Kynurenine | 1.11971 | 4.81925E-06 | 2.42 |
| C63 | (±)8-HDoHE | 1.69331 | 0.000138712 | 2.43 |
| C64 | 9-HOTrE | 1.46044 | 0.000335984 | 2.44 |
| C65 | CPA(18:0/0:0) | 1.77307 | 3.36928E-10 | 2.44 |
| C66 | 5-HETE | 7.29325 | 3.09057E-09 | 2.44 |
| C67 | lysoPC 18:0 | 7.36573 | 1.60985E-10 | 2.48 |
| C68 | LPE 20:3 | 1.11027 | 0.002993164 | 2.49 |
| C69 | 5,12-DiHETE | 8.98015 | 2.85683E-10 | 2.49 |
| C70 | Eicosapentaenoic acid | 2.42223 | 1.99143E-09 | 2.51 |
| C71 | LPE O-16:0 | 2.59717 | 1.00852E-05 | 2.56 |
| C72 | LPC 22:6/0:0 | 3.70361 | 0.000119647 | 2.57 |
| C73 | LPC 16:0 | 28.8309 | 1.60985E-10 | 2.57 |
| C74 | Indoline | 7.46792 | 6.94197E-15 | 2.59 |
| C75 | LPE 18:1 | 3.40854 | 0.008845955 | 2.61 |
| C76 | Phenylalanine | 9.95361 | 2.96884E-14 | 2.62 |
| C77 | FA 18:4+2O/13(S)-HPOT | 1.77635 | 0.007577707 | 2.62 |
| C78 | Prostaglandin B2 | 1.16492 | 0.000111047 | 2.65 |
| C79 | Glycochenodeoxycholic acid 3-glucuronide | 1.74526 | 0.02752 | 2.65 |
| C80 | LPC 20:3 | 1.35602 | 2.63652E-08 | 2.67 |
| C81 | Docosatetraenoic acid | 1.47014 | 4.84541E-07 | 2.68 |
| C82 | (9Z,12R)-12-Hydroxyoctadec-9-enoic acid/5-Hexyltetrahydro-2-furanoctanoic acid | 1.25477 | 2.18652E-06 | 2.69 |
| C83 | LPC O-17:1 | 5.19281 | 1.97529E-11 | 2.74 |
| C84 | 16-Hydroxyhexadecanoic acid/Juniperic acid | 1.54392 | 4.80002E-07 | 2.74 |
| C85 | FA 22:5;O | 2.05537 | 8.8156E-06 | 2.75 |
| C86 | trans-Cinnamic acid | 1.59548 | 5.99658E-15 | 2.77 |
| C87 | LPE 22:5 | 1.08278 | 0.003903647 | 2.78 |
| C88 | FA 20:4;O2/5,6-DiHETE | 4.83819 | 1.92651E-09 | 2.82 |
| C89 | Succinic acid | 1.39259 | 1.65752E-08 | 2.83 |
| C90 | FA 18:2;O/13-HODE | 8.31832 | 3.24753E-07 | 2.86 |
| C91 | LPC 22:4 | 1.68758 | 0.000225505 | 2.87 |
| C92 | Pyroglutamic acid | 3.02453 | 7.89283E-13 | 2.91 |
| C93 | α-Curcumene | 1.03176 | 4.30439E-13 | 2.97 |
| C94 | 8(R)-Hydroxy-(5Z,9E,11Z,14Z)-eicosatetraenoic acid | 1.58288 | 3.66731E-06 | 2.98 |
| C95 | 20-HDoHE | 1.95286 | 7.88913E-05 | 3.00 |
| C96 | LysoPC(O-18:0) | 3.72412 | 5.38074E-08 | 3.08 |
| C97 | Eicosapentaenoic acid | 3.68535 | 1.97529E-11 | 3.10 |
| C98 | FA 16:0(2OH)/2-hydroxyhexadecanoic acid | 4.48124 | 8.38251E-05 | 3.11 |
| C99 | CPA(16:0/0:0) | 1.71523 | 1.97845E-06 | 3.15 |
| C100 | cis-4,7,10,13,16-docosapentaenoic acid | 1.932 | 2.48607E-07 | 3.16 |
| C101 | LPC O-16:1 | 7.51666 | 8.08045E-11 | 3.16 |
| C102 | 5-OXO-D-PROLINE | 4.1297 | 1.7057E-14 | 3.21 |
| C103 | LPA 18:0 | 2.05923 | 2.47799E-10 | 3.22 |
| C104 | 17,18-EpETE | 2.36873 | 3.90008E-12 | 3.24 |
| C105 | Dl-Glutamic acid | 1.57958 | 1.67172E-12 | 3.27 |
| C106 | 9-HODE/FA 18:2;O | 2.92406 | 4.3435E-06 | 3.28 |
| C107 | 2-Ethoxynaphthalene | 1.11045 | 3.48702E-06 | 3.31 |
| C108 | LPC O-18:2 | 3.13032 | 1.17214E-09 | 3.31 |
| C109 | 7-HDoHE | 2.83633 | 0.001159827 | 3.31 |
| C110 | β-linolenic acid | 5.01552 | 5.16469E-07 | 3.45 |
| C111 | 5(S),6(R)-Lipoxin a4 | 1.8399 | 4.43024E-06 | 3.45 |
| C112 | 13-OxoODE | 1.57693 | 0.000165267 | 3.47 |
| C113 | α-Linolenic acid | 1.59083 | 4.80002E-07 | 3.56 |
| C114 | LPC O-18:1 | 8.63961 | 1.94629E-13 | 3.72 |
| C115 | FA 20:3;O/12(S)-HETrE | 2.3674 | 6.16406E-08 | 3.77 |
| C116 | 15-HETE | 3.96687 | 2.45426E-05 | 3.93 |
| C117 | LPI 16:0 | 3.71017 | 4.38306E-05 | 3.96 |
| C118 | LPC 20:1/0:0 | 3.50468 | 5.38074E-08 | 4.02 |
| C119 | LPE 17:0 | 1.18294 | 5.44395E-05 | 4.05 |
| C120 | 15(R)-HEDE | 1.43218 | 3.60002E-07 | 4.07 |
| C121 | LysoPC(0:0/18:0) | 11.5822 | 1.44059E-08 | 4.09 |
| C122 | FA 20:3/Cis-8,11,14-Eicosatrienoic acid | 2.62736 | 1.21482E-08 | 4.09 |
| C123 | FA 20:3;O/8(S)-HETrE | 2.51223 | 1.78435E-05 | 4.13 |
| C124 | ST 24:2;O3;S | 1.23092 | 0.001425658 | 4.13 |
| C125 | LPC O-17:0 | 1.26884 | 1.97037E-10 | 4.15 |
| C126 | 11-HETE | 3.83121 | 1.01223E-06 | 4.17 |
| C127 | LPC 17:0 | 4.94607 | 2.48607E-07 | 4.24 |
| C128 | LPC O-16:0 | 10.4815 | 1.61717E-12 | 4.27 |
| C129 | 13-HOTrE | 2.99132 | 2.42951E-05 | 4.36 |
| C130 | Stearidonic acid | 1.831 | 2.19333E-05 | 4.43 |
| C131 | 17-Hydroxylinolenic acid | 1.06551 | 6.38174E-05 | 4.46 |
| C132 | 15-Oxoete | 1.62475 | 1.00485E-06 | 4.55 |
| C133 | Polyoxyethylene (600) monoricinoleate | 1.74086 | 1.44059E-08 | 4.56 |
| C134 | LPE 16:0 | 6.93618 | 0.000147491 | 4.57 |
| C135 | 8,9-DiHETrE | 2.88359 | 2.65949E-06 | 4.57 |
| C136 | LPE 18:0 | 5.84396 | 2.23883E-07 | 4.86 |
| C137 | 5(S)-HETE | 1.72858 | 3.07945E-08 | 4.95 |
| C138 | LPC 20:2/0:0 | 3.97471 | 1.31067E-07 | 4.95 |
| C139 | LPC 22:5/0:0 | 1.94206 | 3.41678E-06 | 5.02 |
| C140 | LPC O-20:2 | 1.99123 | 7.68458E-10 | 5.13 |
| C141 | FA 20:4;O/12-HETE | 8.7141 | 2.3146E-10 | 5.26 |
| C142 | LPC O-20:1 | 2.26297 | 6.10651E-09 | 5.28 |
| C143 | BA 24:1;O4;G;S/Glycochenodeoxycholate 7-sulfate | 5.05177 | 0.012371242 | 5.29 |
| C144 | 5-OxoETE | 1.11204 | 1.54228E-08 | 5.55 |
| C145 | 16(R)-HETE | 4.25455 | 1.17214E-09 | 6.39 |
| C146 | Arachidonic acid | 9.17545 | 3.17506E-12 | 6.56 |
| C147 | Delta-12-Prostaglandin J2\|Delta-12-PGJ2 | 1.86499 | 1.78536E-05 | 6.72 |
| C148 | LPA 16:0 | 1.52202 | 4.39467E-07 | 8.81 |
| C149 | Pyroglutamyl-Isoleucine | 1.45584 | 5.44395E-05 | 11.05 |
| C150 | 12(S)-HHT | 1.25283 | 1.15561E-05 | 14.38 |
| C151 | Indoleacetic acid | 1.28754 | 2.96156E-13 | 18.29 |
| C152 | Glycerol-2-phosphate | 2.1314 | 6.05182E-19 | 21.22 |
| C153 | Androsterone glucuronide | 1.54879 | 2.08735E-10 | 22.63 |
| C154 | 3-Hydroxyvalproic acid/3-OH-VPA | 1.2964 | 7.02577E-12 | 25.06 |
| C155 | LPA 20:4 | 4.97766 | 4.51269E-08 | 41.65 |
| C156 | 10-Hydroxydecanoic acid | 1.7784 | 1.23869E-08 | 56.26 |
| C157 | FA 8:0 | 1.76381 | 0.001773538 | 85.46 |
| C158 | 2-Hydroxybenzothiazole | 2.45807 | 9.33956E-16 | 98.23 |
| C159 | DG 41:10 | 1.28608 | 1.76003E-11 | 127.09 |
| C160 | 5-Methoxysalicylic acid | 1.77759 | 1.54885E-08 | 162.12 |

**Supplementary Table S3** Regression shrinkage coefficients for 160 differential metabolites via the LASSO.

| **ID** | **Name** | **Coefficients** | **ID** | **Name** | **Coefficients** |
| --- | --- | --- | --- | --- | --- |
| M181T11_1 | Propylparaben | 0 | M331T27 | Docosatetraenoic acid | 0 |
| M239T12 | CMPF | 0 | M297T19_2 | (9Z,12R)-12-Hydroxyoctadec-9-enoic acid /5-Hexyltetrahydro-2-furanoctanoic acid | 0 |
| M539T9_1 | Tetrahydroaldosterone-3-glucuronide | -2.27E-05 | M492T19 | LPC O-17:1 | 0 |
| M541T9_2 | Chaetoglobosin N | 0 | M271T18 | 16-Hydroxyhexadecanoic acid/Juniperic acid | 0 |
| M271T11 | Dehydroepiandrosterone | 0 | M345T20_3 | FA 22:5;O | 0 |
| M188T10 | 3-Indolepropionic acid | 0 | M149T2 | trans-Cinnamic acid | 0 |
| M129T5 | 3-methyl-2-oxovaleric acid | 0 | M526T18 | LPE 22:5 | 0 |
| M367T11_1 | Dehydroepiandrosterone sulfate | 0 | M335T15_2 | FA 20:4;O2/5,6-DiHETE | 0 |
| M129T4 | 3-methyl-2-oxovaleric acid | 0 | M117T1_2 | Succinic acid | 0 |
| M369T12_1 | 5a-Dihydrotestosterone sulfate | 0 | M295T19_1 | FA 18:2;O/13-HODE | 0 |
| M178T5 | Hippurate | 0 | M616T19_1 | LPC 22:4 | 0 |
| M173T4 | Shikimic acid | 0 | M128T1_2 | Pyroglutamic acid | 0 |
| M113T1_2 | Trifluoroacetic acid | 0 | M203T16 | α-Curcumene | 1.09E-05 |
| M447T14_1 | 4-[(2S)-2-hydroxy-3-methyl-3-[(2S,3R,4S,5S,6R)-3,4,5-trihydroxy-6-(hydroxymethyl)oxan-2-yl]oxybutoxy]furo[3,2-g]chromen-7-one | 0 | M319T18 | 8(R)-Hydroxy-(5Z,9E,11Z,14Z)-eicosatetraenoic acid | 0 |
| M162T1_2 | L-Carnitine | -2.35E-06 | M343T19_1 | 20-HDoHE | 0 |
| M140T1 | 3-hydroxy-1,2-dimethylpyridin-4(1H)-one | 0 | M554T22_2 | LysoPC(O-18:0) | 0 |
| M137T1 | Hypoxanthine | 0 | M303T20_2 | Eicosapentaenoic acid | 0 |
| M504T17_2 | LPC17:2 | 0 | M271T23 | FA 16:0(2OH)/ 2-hydroxyhexadecanoic acid | 0 |
| M187T8_2 | Azelaic acid | 0 | M391T18 | CPA(16:0/0:0) | 0 |
| M132T1_2 | Isoleucine | 0 | M329T26 | cis-4,7,10,13,16-docosapentaenoic acid | 0 |
| M253T24_1 | Palmitoleic acid | 0 | M480T19_2 | LPC O-16:1 | 0 |
| M311T19 | 8(R)-HPODE | 0 | M128T1_1 | 5-OXO-D-PROLINE | 0 |
| M97T1_2 | Phosphoric acid | 0 | M437T21 | LPA 18:0 | 0 |
| M257T26 | Butyl dodecanoate | 0 | M301T16 | 17,18-EpETE | 0 |
| M279T25_1 | Linoleic acid | 0 | M130T1_1 | Dl-Glutamic acid | 3.06E-06 |
| M327T24_1 | Docosahexanoic acid | 0 | M295T18 | 9-HODE/FA 18:2;O | 0 |
| M147T1_2 | Lysine | 0 | M173T17_1 | 2-Ethoxynaphthalene | 0 |
| M498T12_2 | taurochenodeoxycholic acid | 0 | M506T20_1 | LPC O-18:2 | 0 |
| M103T2 | 2-Hydroxybutyric acid | 0 | M343T19_2 | 7-HDoHE | 0 |
| M359T16_3 | Resolvin D5 | 0 | M279T19_1 | β-linolenic acid | 0 |
| M450T20_3 | LPE O-17:1 | 0 | M351T13_4 | 5(S),6(R)-Lipoxin a4 | 0 |
| M297T18_1 | C10-LAS (TENTATIVE) | 0 | M293T20_1 | 13-OxoODE | 0 |
| M333T14_1 | LTB5 | 0 | M279T18 | α-Linolenic acid | 0 |
| M619T18_1 | LPI 20:4 | 0 | M508T19 | LPC O-18:1 | 0 |
| M331T21_2 | (5Z,8Z,11Z,14Z,17Z)-Eicosapentaenoic acid ethyl ester | 0 | M321T20_1 | FA 20:3;O/12(S)-HETrE | 0 |
| M500T17_2 | LPE 20:4 | 0 | M319T19_2 | 15-HETE | 0 |
| M414T14_1 | glycoursodeoxycholic acid | 0 | M571T19 | LPI 16:0 | 0 |
| M375T12_4 | Resolvin D3/  FA 22:6,O3 | 0 | M550T21_3 | LPC 20:1/0:0 | 0 |
| M261T23_2 | NCGC00381425-01!8-hydroxy-8-(3-octyloxiran-2-yl)octanoic acid | 0 | M466T19_1 | LPE 17:0 | 0 |
| M676T30 | SM 32:1;O2 | 0 | M323T21 | 15(R)-HEDE | 0 |
| M343T20_2 | 10-HDoHE | 0 | M524T20 | LysoPC(0:0/18:0) | 0 |
| M438T19_1 | LPE O-16:1 | 0 | M305T26 | FA 20:3/Cis-8,11,14-Eicosatrienoic acid | 0 |
| M704T27_1 | SM 34:1;O2 | 0 | M321T20_2 | FA 20:3;O/8(S)-HETrE | 0 |
| M566T19_2 | LPC 18:1 | 0 | M469T11_2 | ST 24:2;O3;S | 0 |
| M255T26 | hexadecanoic acid/  FA 16:0 | 0 | M496T20 | LPC O-17:0 | 0 |
| M333T18_2 | FA 20:5;O2 | 0 | M319T19_1 | 11-HETE | 0 |
| M432T14_2 | Glycochenodexycholate | 0 | M510T19_1 | LPC 17:0 | 0 |
| M781T30 | PC 36:5 | 0 | M482T19 | LPC O-16:0 | 0 |
| M464T19 | LPE O-18:2 | 0 | M293T19_1 | 13-HOTrE | 0 |
| M229T1 | Pro-Leu | 0 | M277T19 | Stearidonic acid | 0 |
| M104T1_1 | Choline | 2.37E-07 | M295T20 | 17-Hydroxylinolenic acid | 0 |
| M466T22 | LPE O-18:1 | 0 | M317T20 | 15-Oxoete | 0 |
| M297T19_3 | FA 18:1+1O | 0 | M341T21_3 | Polyoxyethylene (600) monoricinoleate | 0 |
| M506T19_2 | LPC 17:1 | 0 | M452T18_1 | LPE 16:0 | 0 |
| M380T16 | Sphinganine 1-phosphate\|Dihydrosphingosine 1-phosphate | 0 | M337T15 | 8,9-DiHETrE | 0 |
| M351T12_3 | 5(S),6(R),15(R)-Lipoxin A4 | 0 | M482T21_1 | LPE 18:0 | 0 |
| M329T11_2 | (Z)-5,8,11-trihydroxyoctadec-9-enoic acid | 0 | M303T19_2 | 5(S)-HETE | 0 |
| M779T26 | PC 36:6 | 0 | M548T20 | LPC 20:2/0:0 | 0 |
| M280T22 | Linoleamide | 0 | M570T19 | LPC 22:5/0:0 | 0 |
| M307T27 | FA 20:2/ Cis-11,14-Eicosadienoic acid | 0 | M534T20_1 | LPC O-20:2 | 0 |
| M311T18_1 | FA 18:2+2O/ 11-HpODE | 0 | M319T20_2 | FA 20:4;O/12-HETE | 0 |
| M209T2 | Kynurenine | 0 | M536T22 | LPC O-20:1 | 0 |
| M343T20_1 | (±)8-HDoHE | 0 | M528T12_2 | BA 24:1;O4;G;S/ Glycochenodeoxycholate 7-sulfate | 0 |
| M293T17_2 | 9-HOTrE | 0 | M319T21 | 5-OxoETE | 0 |
| M419T21 | CPA(18:0/0:0) | 0 | M303T20_3 | 16(R)-HETE | 0 |
| M319T20_1 | 5-HETE | 0 | M305T25 | Arachidonic acid | 0 |
| M552T19_2 | lysoPC 18:0 | 0 | M333T17_2 | Delta-12-Prostaglandin J2\|Delta-12-PGJ2 | 0 |
| M502T18 | LPE 20:3 | 0 | M409T26 | LPA 16:0 | 0 |
| M335T16_3 | 5,12-DiHETE | 0 | M241T7 | Pyroglutamyl-Isoleucine | 0 |
| M301T20_2 | Eicosapentaenoic acid | 0 | M279T17_1 | 12(S)-HHT | 0 |
| M438T19_3 | LPE O-16:0 | 0 | M174T8 | Indoleacetic acid | 0 |
| M568T17 | LPC 22:6/0:0 | 0 | M171T1 | Glycerol-2-phosphate | 5.85E-05 |
| M496T18_2 | LPC 16:0 | 2.44E-08 | M467T20 | Androsterone glucuronide | 0 |
| M120T2 | Indoline | 0 | M159T8 | 3-Hydroxyvalproic acid/3-OH-VPA | 0 |
| M478T19 | LPE 18:1 | 0 | M457T21_2 | LPA 20:4 | 0 |
| M166T2 | Phenylalanine | 0 | M187T13_2 | 10-Hydroxydecanoic acid | 0 |
| M309T17_2 | FA 18:4+2O/ 13(S)-HPOT | 0 | M143T13 | FA 8:0 | 0 |
| M333T14_2 | Prostaglandin B2 | 0 | M150T26 | 2-Hydroxybenzothiazole | 4.19E-06 |
| M624T12_2 | Glycochenodeoxycholic acid 3-glucuronide | 0 | M697T18 | DG 41:10 | 2.88E-06 |
| M568T18_2 | LPC 20:3 | 0 | M169T16 | 5-Methoxysalicylic acid | 0 |

**Supplementary Table S4** Demographics and clinical characteristics of low activity DM and high activity DM.

| Characteristic | disease activity of DM | | P value |
| --- | --- | --- | --- |
|  | Low activity DM (n=40) | High activity DM (n=55) |  |
| Sex, female (%) | 33 (82.5%) | 44(80.0%) |  |
| Age (years) | 46.05±15.02 | 51.27±15.48 |  |
| BMI (kg/m2, mean ± SD) | 24.16±6.38 | 23.15±4.58 |  |
| Age at onset | 42.28±15.41 | 49.13±15.91 |  |
| Disease duration (median mouth, IQR) | 11 (2, 33) | 5 (1.5, 16) |  |
| LDH, U/L | 417.18±205.33 | 520.47±424.12 |  |
| Cr, μmol/L | 50.20±11.45 | 47.63±11.74 |  |
| CK, μmol/L | 49.84 (27.05, 90.88) | 59.50 (23.75, 403.5) |  |
| AST, U/L | 62.01±65.11 | 100.49±121.18 |  |
| ALT, U/L | 65.09±104.99 | 88.84±125.01 |  |
| TC, mM/L | 4.67±1.32 | 4.37±0.99 |  |
| TG, mM/L | 1.89±0.73 | 1.87±1.17 |  |
| HDL-C, mmol/L | 1.22±0.43 | 1.09±0.30 |  |
| LDL-C, mmol/L | 2.66±0.99 | 2.43±0.59 |  |
| Glucose, mmol/L | 5.42±1.46 | 5.98±2.07 |  |
| ESR, mm/h | 38.89±28.26 | 47.46±30.06 |  |
| CRP, mg/ | 23.70±29.58 | 34.38±55.07 |  |
| ANA(1:80), (n, %) | 21 (52.5%) | 28 (50.91%) |  |
| MYOACT | 6.35±2.36 | 14.06±3.36 | <0.01 |
| **Skin lesions, n (%)** |  |  |  |
| Gottron’s papules or sign | 7 (17.5%) | 32 (58.18%) | <0.01 |
| Heliotrope rash | 33 (82.5%) | 51 (92.73%) |  |
| Mechanics hand | 1 (2.5%) | 8 (14.54%) | 0.049 |
| Cutaneous ulcerations | 2 (5%) | 9 (16.36%) |  |
| **Muscle, n (%)** |  |  |  |
| Myalgia | 13 (32.5%) | 25 (45.45%) |  |
| Muscle weakness | 24 (60%) | 51 (92.73%) | <0.01 |
| **Other, n (%)** |  |  |  |
| Hoarseness or sore throat, dysphagia | 13 (32.5%) | 33 (60%) | 0.08 |
| Arthralgia | 11 (27.5%) | 38 (69.09%) |  |
| Fever | 21 (52.5%) | 22 (40%) |  |
| Cardiac involvement | 2 (5%) | 8 (14.54%) |  |
| **Myositis-specific antibodies, no. (%)** | | | |
| MDA-5 | 2 (5%) | 9 (16.36%) |  |
| TIF-1γ | 2 (5%) | 6 (10.91%) |  |
| Jo-1 | 1 (2.5%) | 5 (9.09%) |  |
| Mi-2 | 1 (2.5%) | 5 (9.09%) |  |
| NXP-2 | 1 (2.5%) | 4 (7.27%) |  |
| Ro52 | 7 (17.5%) | 13 (23.64%) |  |
| **Lymphocyte subpopulation** | | | |
| T cell (CD3+CD19-) (/μL) | 1134±759 | 799±711 | 0.05 |
| B cell (CD3-CD19+) (/μL) | 265±233 | 235±283 |  |
| Th cell (CD3+CD4+) (/μL, IQR) | 621±447 | 443±362 | 0.01 |
| CD8+T cell (CD3+CD8+)(/μL) | 474±468 | 328±465 | 0.016 |
| Th cell/CD8+T cell | 1.87±1.31 | 1.81±1.17 |  |
| NK cell (CD3-, CD56+) (/μL) | 148±114 | 114±81 |  |
| **CD4+ T cell subsets** | | | |
| Th1(/μL) | 60±81 | 47.16±65.11 |  |
| %Th1 | 8.58±7.72 | 10.16±9.98 |  |
| Th2(/μL) | 8.51±8.67 | 6.17±5.98 |  |
| %Th2 | 1.29±0.79 | 1.67±1.96 |  |
| Th17(/μL) | 7.03±8.61 | 4.71±4.73 |  |
| %Th17 | 0.99±0.81 | 1.37±1.92 |  |
| Treg(/μL) | 32.46±24.50 | 24.50±23.28 | 0.027 |
| %Treg | 5.44±2.83 | 5.21±2.12 |  |
| Th1/Th2 | 7.33±5.87 | 10.47±11.26 |  |
| Th17/Treg | 0.27±0.39 | 0.29±0.30 |  |

**Supplementary Table S5** Demographics and clinical characteristics of DM with ILD and DM without ILD.

| Characteristic | DM with or without interstitial lung disease（ILD） | |  |
| --- | --- | --- | --- |
|  | DM-ILD (n=36) | Without ILD (n=59) | P value |
| Sex, female (%) | 28 (77.78%) | 49 (83.05%) |  |
| Age (years) | 54.11±11.88 | 46±16.69 |  |
| BMI (kg/m2, mean ± SD) | 24.37±3.09 | 23.07±6.45 |  |
| Age at onset | 51.94±11.61 | 42.76±17.33 |  |
| Disease duration (median mouth, IQR) | 9 (2, 19) | 6 (1, 31.5) |  |
| LDH, U/L | 419.33±282.13 | 510.44±383.59 |  |
| Cr, μmol/L | 48.53±10.29 | 48.83±12.43 |  |
| CK, μmol/L | 62.99 (30.50, 233.74) | 44.68 (20.50, 296) |  |
| AST, U/L | 69.17±59.33 | 92.98±120.74 |  |
| ALT, U/L | 66.99±65.31 | 85.70±138.86 |  |
| TC, mM/L | 4.80±1.38 | 4.31±0.93 |  |
| TG, mM/L | 2.18±1.30 | 1.71±0.77 |  |
| HDL-C, mmol/L | 1.19±0.35 | 1.11±0.37 |  |
| LDL-C, mmol/L | 2.68±0.87 | 2.43±0.71 |  |
| Glucose, mmol/L | 5.91±1.98 | 5.86±2.38 |  |
| ESR, mm/h | 38 (23.25, 57.50) | 33.5 (21.75, 75) |  |
| CRP, mg/ | 10.07 (3.79, 18.43) | 12.20 (3.87, 49.80) |  |
| ANA(1:80), (n, %) | 24 (66.67%) | 25 (42.37%) | 0.027 |
| MYOACT | 20.86±8.77 | 17.56±8.42 |  |
| **Skin lesions, n (%)** |  |  |  |
| Gottron’s papules or sign | 19 (52.78%) | 20 (33.90%) |  |
| Heliotrope rash | 32 (88.89%) | 52 (88.14%) |  |
| Mechanics hand | 5 (13.89%) | 4 (6.78%) |  |
| Cutaneous ulcerations | 4 (11.11%) | 7 (11.86%) |  |
| **Muscle, n (%)** |  |  |  |
| Myalgia | 13 (36.11%) | 25 (42.37%) |  |
| Muscle weakness | 29 (80.56%) | 46 (77.97%) |  |
| **Other, n (%)** |  |  |  |
| Hoarseness or sore throat, dysphagia | 21 (58.33%) | 25 (42.37%) |  |
| Arthralgia | 19 (52.78%) | 30 (50.85%) |  |
| Fever | 15 (41.67%) | 28 (47.46%) |  |
| Cardiac involvement | 7 (19.44%) | 3 (5.08%) | 0.028 |
| **Myositis-specific antibodies, no. (%)** | | | |
| MDA-5 | 9 (25%) | 2 (3.39%) | 0.001 |
| TIF-1γ | 5 (13.89%) | 3 (5.08%) |  |
| Jo-1 | 4 (11.11%) | 2 (3.39%) |  |
| Mi-2 | 2 (5.56%) | 1 (1.69%) |  |
| NXP-2 | 2 (5.56%) | 3 (5.08%) |  |
| Ro52 | 18 (50%) | 2 | 0.001 |
| **Lymphocyte subpopulation** | | | |
| T cell (CD3+CD19-) (/μL) | 752±766 | 1056±718 | 0.011 |
| B cell (CD3-CD19+) (/μL) | 217±209 | 266±290 |  |
| Th cell (CD3+CD4+) (/μL, IQR) | 387±267 | 598±457 | 0.032 |
| CD8+T cell (CD3+CD8+)(/μL) | 359±592 | 409±382 | 0.033 |
| Th cell/CD8+T cell | 1.87±1.35 | 1.81±1.17 |  |
| NK cell (CD3-, CD56+) (/μL) | 125±97 | 131±98 |  |
| **CD4+ T cell subsets** |  |  |  |
| Th1(/μL) | 44.26±72.92 | 57.14±72.05 |  |
| %Th1 | 8.37±6.73 | 10.17±10.23 |  |
| Th2(/μL) | 6.86±8.66 | 7.32±6.45 |  |
| %Th2 | 1.85±2.33 | 1.32±0.90 |  |
| Th17(/μL) | 5.66±8.31 | 5.69±5.67 |  |
| %Th17 | 1.55±2.39 | 1.02±0.71 |  |
| Treg(/μL) | 22.19±14.42 | 31.01±27.59 |  |
| %Treg | 5.64±2.66 | 5.11±2.28 |  |
| Th1/Th2 | 8.29±7.62 | 9.69±10.47 |  |
| Th17/Treg | 0.34±0.46 | 0.25±0.24 |  |

**Supplementary Table S6.** Demographics and clinical characteristics of DM with different antibody positive (MDA5+DM, TIF1-γ+DM, Jo-1+DM and antibody negative DM (control)).

| Characteristic | Negative(n=21) | MDA-5(+) (n=10) | TIF1-γ(+) (n=8) | Jo-1(+) (n=6) |
| --- | --- | --- | --- | --- |
| Sex, female n (%) | 18 (85.71%) | 6 (60%) | 7 (87.5%) | 5 (83.33%) |
| Age (years) | 39.29±13.67 | 49.70±12.61 | 51.12±17.03 | 61.67±12.60 |
| BMI | 22.72±3.34 | 24.25±3.60 | 22.93±3.40 | 23.42±4.09 |
| Age at onset | 35.43±12.72 | 49.10±12.49 | 50.25±16.83 | 53.33±13.53 |
| Disease duration (median mouth, IQR) | 7 (1, 36) | 3.5 (2, 9) | 6 (1, 10.75) | 90 (51, 138) |
| LDH, U/L | 442.43±233.82 | 364.93±71.73 | 306.18±113.63 | 666.45±406.92 |
| CK, μmol/L | 36.12±25.38 | 98.55±93.43 | 1305.31±3340.12 | 2699.97±3293.36 |
| AST, U/L | 75.99±87.00 | 90.98±66.86 | 58.24±59.95 | 115.13±98.76 |
| ALT, U/L | 124.94±206.85 | 82.88±56.91 | 39.28±19.07 | 96.13±100.81 |
| ESR, mm/h | 40.35±28.66 | 38.00±17.71 | 37.5±36.75 | 32.33±15.21 |
| CRP, mg/ | 33.88±41.89 | 7.83±8.22 | 18.37±41.30 | 21.66±21.02 |
| ANA(1:80), (n, %) | 3 (14.29%) | 5 (50%) | 6 (75%) | 5 (83.33%) |
| MYOACT | 9.86±4.90 | 13.40±5.09 | 12.81±4.92 | 13.25±5.0 |
| **Skin lesions, n (%)** |  |  |  |  |
| Gottron’s papules or sign | 6 (28.57%) | 7 (70%) | 1 (12.5%) | 4 (66.67%) |
| Heliotrope rash | 18 (85.71%) | 9 (90%) | 7 (87.5%) | 2 (33.33%) |
| Mechanics hand | 1 (4.76%) | 3 (30%) | 1 (12.5%) | 0 |
| Cutaneous ulcerations | 3 (14.29%) | 5 (50%) | 1 (12.5%) | 1(16.67%) |
| **Muscle, n (%)** |  |  |  |  |
| Myalgia | 12 (57.14%) | 4 (40%) | 2 (25%) | 1 (16.67%) |
| Muscle weakness | 16 (76.19%) | 7 (70%) | 7 (87.5%) | 5 (83.33%) |
| **Other, n (%)** |  |  |  |  |
| Hoarseness or sore throat, dysphagia | 13 (61.9%) | 7 (70%) | 1 (12.5%) | 3 (50%) |
| Arthralgia | 13 (61.9%) | 7 (70%) | 4 (50%) | 6 (100%) |
| Fever | 14 (66.67%) | 5 (50%) | 3 (37.5%) | 1 (16.67%) |
| Interstitial lung disease | 3 (14.29%) | 7 (70%) | 4 (50%) | 4 (66.67%) |
| Cardiac involvement | 1 (4.76%) | 3 (30%) | 0 | 1 (16.67%) |
| **Lymphocyte subpopulation** | | | | |
| T cell (CD3+CD19-)(median/μL, IQR) | 1303 (692, 2021) | 335 (280, 518) | 488 (386, 621) | 766 (700, 850) |
| B cell (CD3-CD19+)(median/μL, IQR) | 137 (88, 258) | 111 (83, 160) | 158 (111, 256) | 125 (72, 208) |
| Th cell (CD3+CD4+) (median/μL, IQR) | 573 (443, 1096) | 183 (169, 345) | 309 (243, 368) | 491 (422, 555) |
| CD8+T cell (CD3+CD8+)( median/μL, IQR) | 489 (192, 663) | 138 (94, 179) | 155 (78, 305) | 301 (202, 394) |
| NK cell (CD3-, CD56+) (median/μL, IQR) | 97 (72, 140) | 52 (33, 73) | 130 (69, 132) | 224 (168, 284) |
| **CD4+ T cell subsets** | | | | |
| Th1( median/μL, IQR) | 44.65 (16.87, 119.16) | 6.28 (5.86,14.75) | 14.43 (9.67, 27.86) | 46.7 (22.27, 70.26) |
| %Th1 | 5.48 (4.28, 16.71) | 3.65 (3.08,4.82) | 4.83 (2.75, 6.96) | 10.59 (5.63,12.88) |
| Th2( median/μL, IQR) | 8.03 (3.65,11.71) | 3.63 (1.05,3.72) | 7.57 (4.41,12.87） | 4.4 (2.78,5.11) |
| %Th2 | 1.16 (0.94,1.89) | 1.03 (1, 1.21) | 1.75 (1.27,2.94) | 1.01 (0.59,1.18) |
| Th17( median/μL, IQR) | 4.73 (2.55,13.02) | 1.74 (1.37, 5.8) | 3.54 (2.57,4.37) | 2.65 (1.70,4.63) |
| %Th17 | 0.96 (0.52,1.47) | 1.03 (0.54,1.67) | 1.02 (0.66, 1.07) | 0.6 (0.38,0.85) |
| Treg( median/μL, IQR) | 31 (18.77,50.97) | 15.20 (13.91,23.35) | 28.66 (15.98,42.85） | 17.71 (16.39,19.29) |
| %Treg | 4.42 (3.69,5.53) | 7.26 (6.39,8.32) | 4.52 (4.36, 9.70) | 3.86 (3.11,5.47) |
| Th1/Th2 | 5.62 (3.41,13.21) | 4.07 (1.97,6.00) | 2.76 (1.22, 3.71) | 9.43 (6.79,16.26) |
| Th17/Treg | 0.19 (0.08,0.34) | 0.14 (0.07,0.22) | 0.14 (0.10, 0.23) | 0.13(0.10,0.16) |


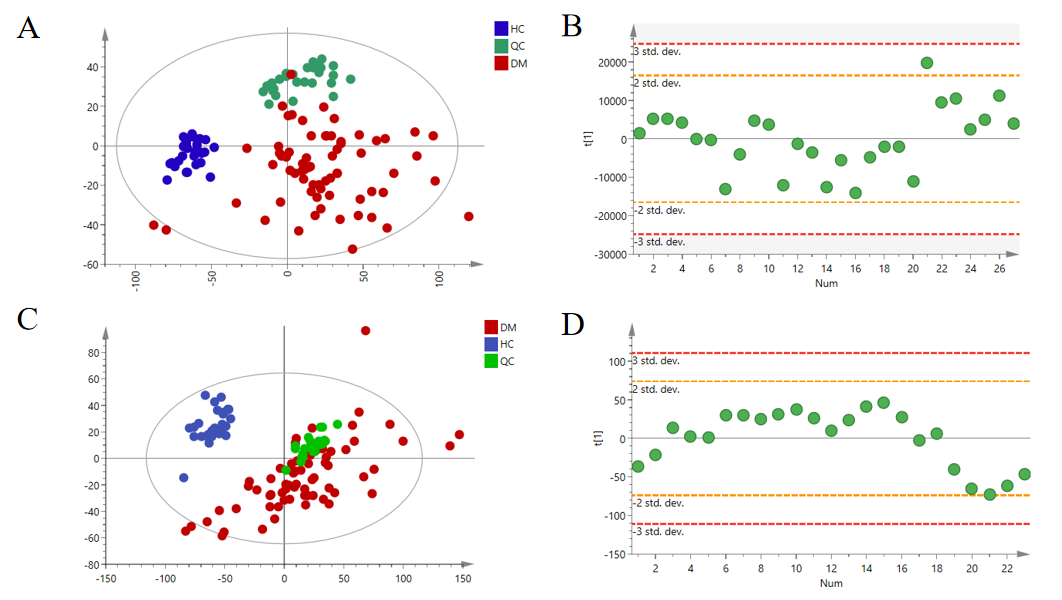


**Supplementary Figure S1** The PCA score plot of discovery set in the positive **(A)** and negative ion mode **(C)**. The PCA line score plots of quality control (QC) samples in positive **(B)** and negative ion mode **(D)**. HC group (blue circle), DM group (red circle), and QC samples (green circle).


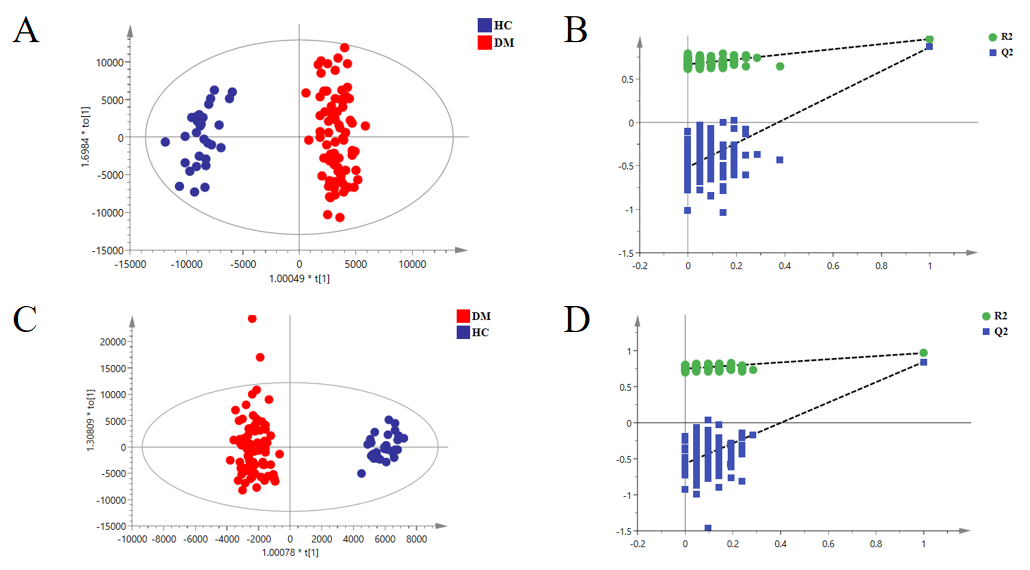


**Supplementary Figure S2** Analysis of serum differential metabolites between DM and HCs. OPLS-DA score scatter plots of discovery cohort between HC group and DM group in positive **(A)** and negative ion modes **(C)**. The result of permutation test in positive ion mode **(B)** and negative ion mode **(D)**.


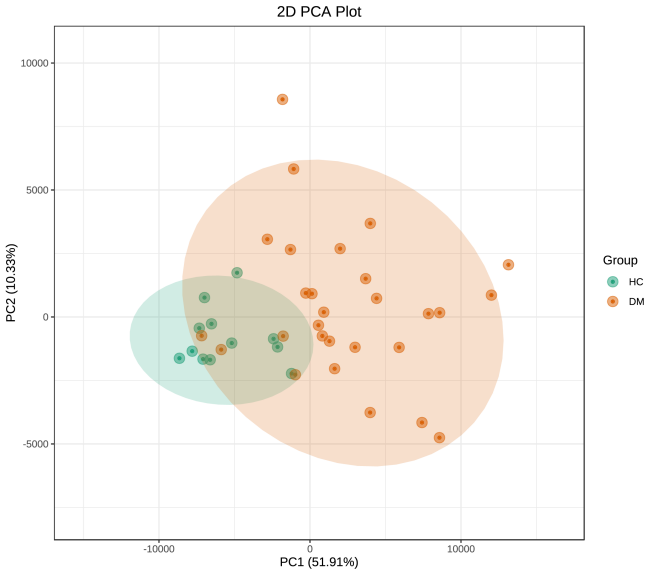


**Supplementary Figure S3** The PCA score plot of 160 differential metabolites based on HCs and patients with DM in the validation set.


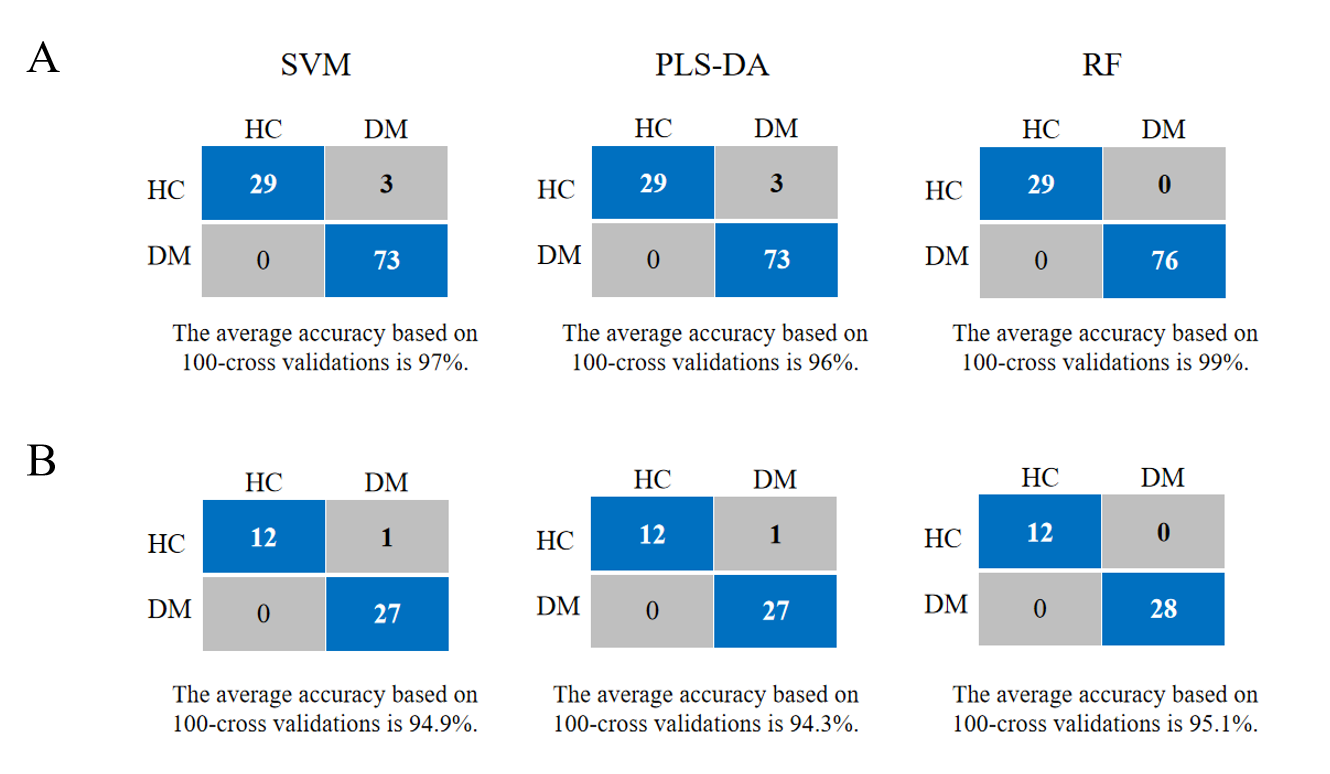


**Supplementary Figure S4** Machine learning models predicted the classification accuracy of biomarkers. **(A)** The discrimination ability of two biomarkers was verified by SVM, PLS-DA and RF models in the discovery cohort. **(B)** The discrimination ability of two biomarkers was verified by SVM, PLS-DA and RF models in the validation cohort.


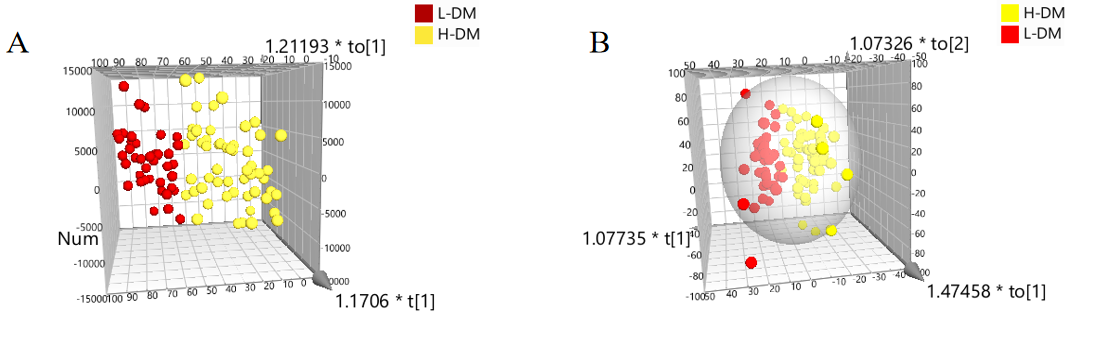


**Supplementary Figure S5** Analysis of serum differential metabolites between low activity DM (L-DM) and high activity DM (H-DM). Metabolites profiling analysis in the positive (**A**) and negative ion mode (**B**) for human serum OPLS-DA score plots from the L-DM and H-DM.


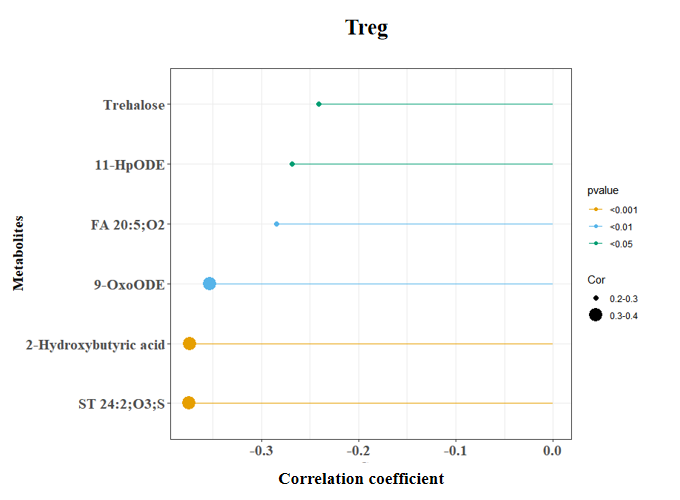


**Supplementary Figure S6** Correlation between differential metabolites (H-DM vs L-DM) and absolute value of Treg.


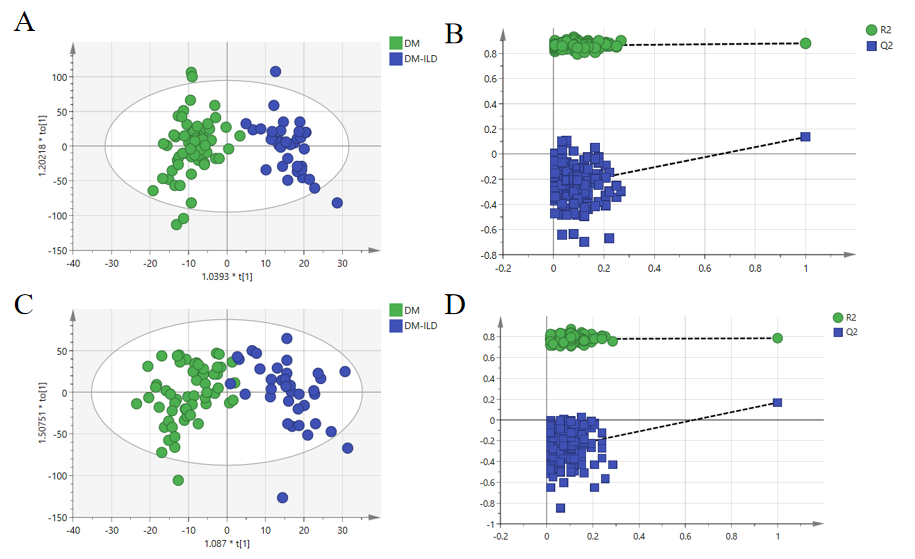


**Supplementary Figure S7** OPLS-DA score scatter plots of discovery set between DM-ILD and DM without ILD in positive (**A**) and negative ion mode (**C**). The result of permutation test in positive ion mode (**B**) and negative ion mode (**D**).


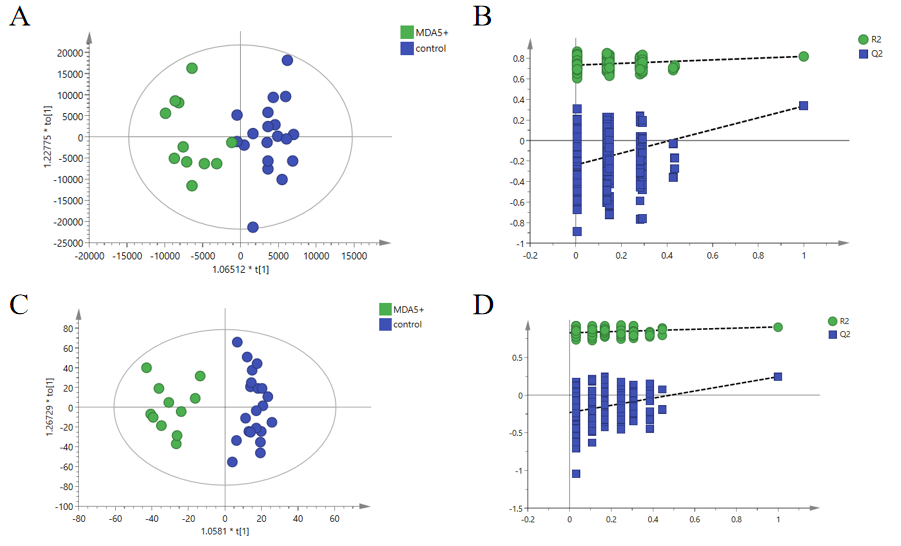
**Supplementary Figure S8** OPLS-DA score scatter plots of discovery set between MDA5+DM and antibody negative DM (control) in positive **(A)** and negative ion mode **(C)**. The result of permutation test in positive ion mode **(B)** and negative ion mode **(D)**.


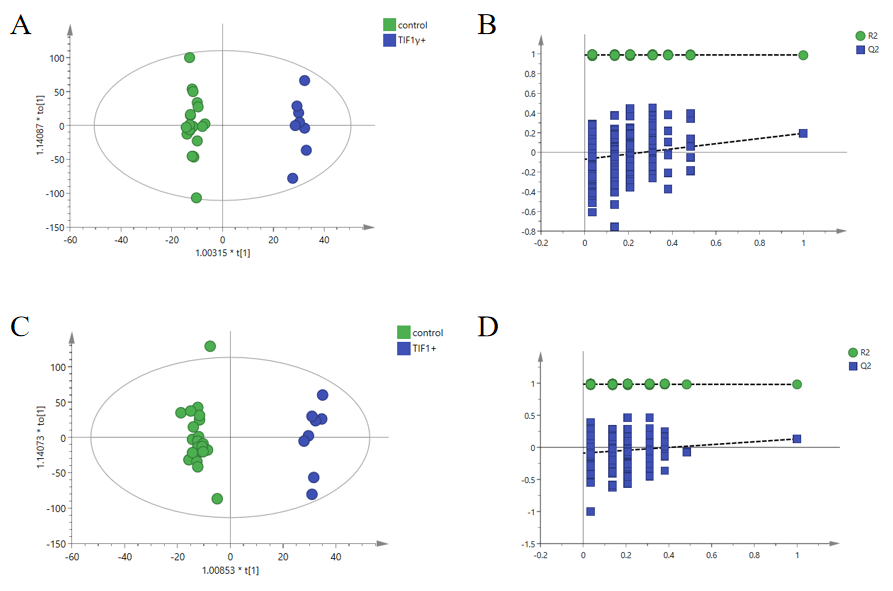


**Supplementary Figure S9** OPLS-DA score scatter plots of discovery set between TIF1-γ+DM and antibody negative DM (control) in positive (**A**) and negative ion mode (**C**). The result of permutation test in positive ion mode (**B**) and negative ion mode (**D**).


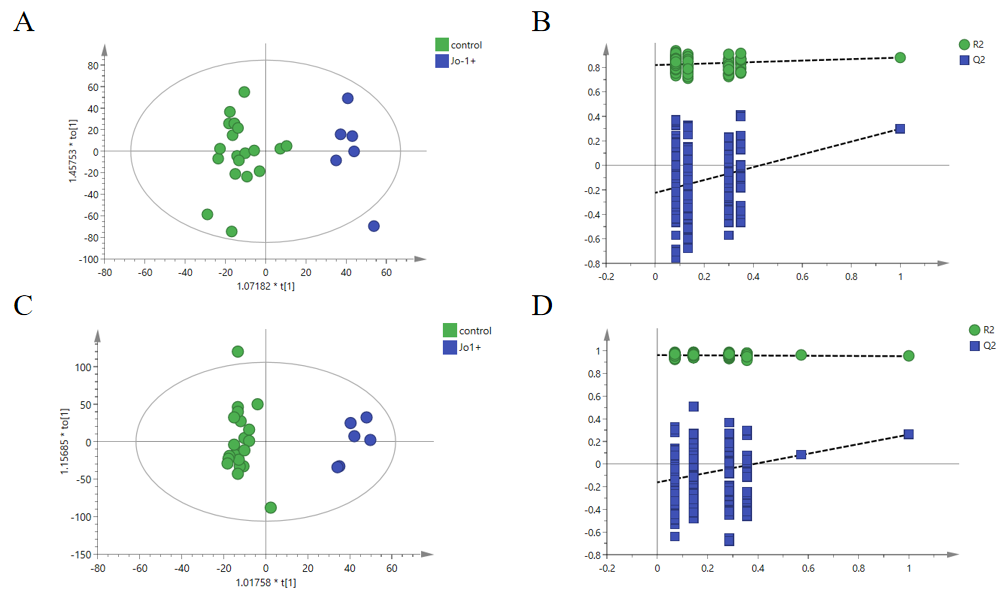


**Supplementary Figure S10** OPLS-DA score scatter plots of discovery set between Jo-1+DM and antibody negative DM (control) in positive (**A**) and negative ion mode (**C**). The result of permutation test in positive ion mode (**B**) and negative ion mode (**D**).


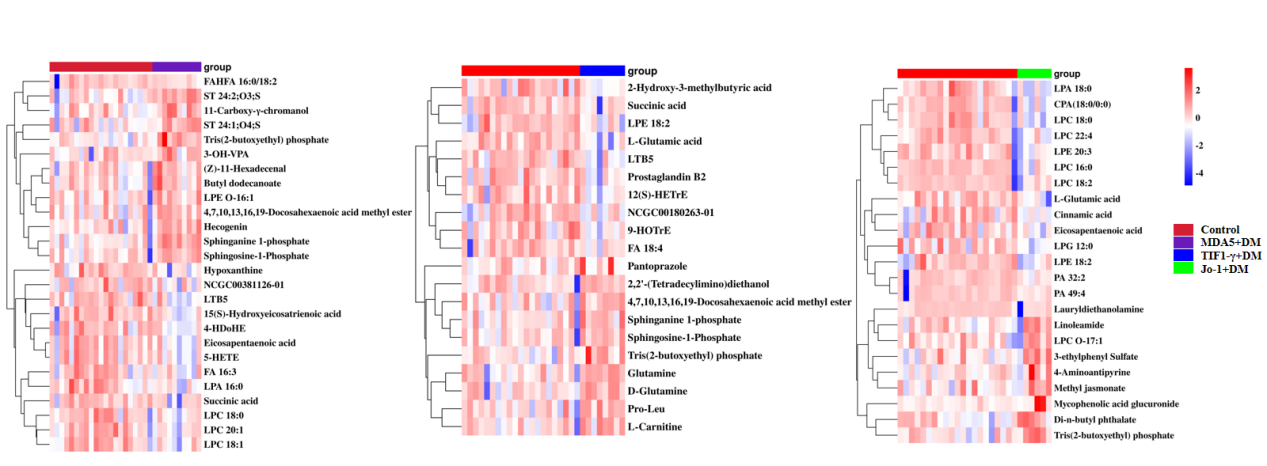


**Supplementary Figure S11** Heatmaps of differential metabolites between MDA5+DM, TIF1-γ+DM, and Jo-1+DM and control, respectively.
